# Doped Zinc Oxide Nanoparticles: Synthesis, Characterization and Potential Use in Nanomedicine

**DOI:** 10.3390/app10155194

**Published:** 2020-07-28

**Authors:** Marco Carofiglio, Sugata Barui, Valentina Cauda, Marco Laurenti

**Affiliations:** Department of Applied Science and Technology, Politecnico di Torino, Corso Duca degli Abruzzi 24, 10129 Turin, Italy

**Keywords:** zinc oxide nanoparticles, doped ZnO, rare earth, transition metals, bioimaging, drug delivery, theranostics, antibacterial properties

## Abstract

Smart nanoparticles for medical applications have gathered considerable attention due to an improved biocompatibility and multifunctional properties useful in several applications, including advanced drug delivery systems, nanotheranostics and in vivo imaging. Among nanomaterials, zinc oxide nanoparticles (ZnO NPs) were deeply investigated due to their peculiar physical and chemical properties. The large surface to volume ratio, coupled with a reduced size, antimicrobial activity, photocatalytic and semiconducting properties, allowed the use of ZnO NPs as anticancer drugs in new generation physical therapies, nanoantibiotics and osteoinductive agents for bone tissue regeneration. However, ZnO NPs also show a limited stability in biological environments and unpredictable cytotoxic effects thereof. To overcome the abovementioned limitations and further extend the use of ZnO NPs in nanomedicine, doping seems to represent a promising solution. This review covers the main achievements in the use of doped ZnO NPs for nanomedicine applications. Sol-gel, as well as hydrothermal and combustion methods are largely employed to prepare ZnO NPs doped with rare earth and transition metal elements. For both dopant typologies, biomedical applications were demonstrated, such as enhanced antimicrobial activities and contrast imaging properties, along with an improved biocompatibility and stability of the colloidal ZnO NPs in biological media. The obtained results confirm that the doping of ZnO NPs represents a valuable tool to improve the corresponding biomedical properties with respect to the undoped counterpart, and also suggest that a new application of ZnO NPs in nanomedicine can be envisioned.

## 1 Introduction

Nanomedicine is an emerging branch of science dealing with the use of smart materials for medical applications. Some practical examples include new generation functional materials for physical therapy (hyperthermia, photodynamics and sonodynamics) and advanced drug delivery systems, but also for tissue engineering, contrast agents and in vivo imaging.

The integration of smart materials with nanomedicine is challenging as it poses a severe limitation about the corresponding size, which must be comparable to that characterizing most of the biological systems. For this purpose, nanomaterials are the most promising candidates to meet such requirements as the average size lies at the nanometer scale. Moreover, the medical technologies resulting from the use of nanomaterials benefit from this reduced size, as they are considered to be less invasive and possibly implantable in the body. Another advantage is that the physical–chemical properties of nanomaterials can be easily tuned with various methods so that unprecedented functionalities with respect to the bare material can be provided. In this sense, nanomaterials for nanomedicine are conceived as smart, i.e., materials designed for the exploitation of specific functions, even remotely controlled and activated by an external stimulus [1]. This specific function can thus be exploited after the injection of the nanomaterial in the human body and only once the site of interest has been reached.

Among nanomedicine applications, the efficacy of nanoparticles-based drug delivery systems has been largely demonstrated [2,3]. With respect to the standard methods, the use of nanoparticles with a high surface area as drug carriers has highly improved the encapsulation efficiency of drugs and a more targeted delivery of the drug to the site of interest is foreseen. Moreover, drug delivery systems based on the use of nanoparticles also show improved pharmacokinetics and biodistribution of the drug [2]. Complex delivery mechanisms have been developed to achieve the controlled release of the drug, including pH-triggered release systems [4] or are driven by an external activation with light/mechanical stimulation [5,6]. In some cases, nanoparticles have been also investigated as nanoantibiotics due to their promising antimicrobial activity [7] and high potential in fighting antibiotic resistance [8]. More recently, nanoparticles started to be studied on their own as new generation nanodrugs for anticancer therapy [9]. Actually, the conventional treatments based on chemotherapy show several limitations: for example, the lack of selectivity and solubility of the chemotherapeutic agents, which induced undesirable side effects [10]. On the other side, the successful treatment of cancer cells by using nanoparticles can benefit from the following aspects: (i) the high surface area to volume ratio and (ii) the rich surface chemistry, both of which allow the anchoring of specific functional groups and make the functionalized nanoparticles more selective toward selected tumor cells [11,12]; (iii) the reduced size (5–100 nm), allowing their preferential accumulation at tumor sites that lack an effective lymphatic drainage system [13–16]; (iv) a spontaneous biodegradation into non-toxic reaction products, thus avoiding the accumulation of toxic elements in the body once the therapeutic action has been completed [17,18].

Nanoparticles with improved functionalities have been developed for in vivo imaging as well [19]. The reduction of the particle size down to the nanometer scale results in quantum confinement effects such as size-tunable light emission properties (quantum dots, QDs). In this sense, nanoparticles can be used as contrast agents with improved imaging properties. For example, fluorescent nanoparticles based on semiconducting QDs, or polymeric or metallic nanoparticles have been developed to achieve higher contrast imaging than using standard molecular fluorophores [20,21]. Magnetic nanoparticles have also been combined with the magnetic resonance imaging (MRI) technique and used as contrast agents for tumor visualization [22]. Metallic nanoparticles have been successfully tested as contrast agents for optical or X-ray imaging and allowed for the detection of atherosclerotic plaques, intravascular thrombus, or fibrotic tissue [23].

More recently, theranostic nanoparticles have captured considerable attention as well [24]. Theranostics deals with the development of multifunctional platforms able to perform diagnostics and therapy at the same time, also with the aid of nanoparticles and nanotechnologies (nanotheranostics). Some materials are characterized by the coexistence of specific physical and chemical properties, such as the optical, magnetic or catalytic properties, so that both the imaging and therapeutic effects are combined together within the same nanoparticles. Within this scenario, it is thus possible to prepare nanosized materials able to be injected in the human body, reach the specific target organ and release the therapeutic agent, but also be directly monitored and visualized in the target site. As an example, Au nanoparticles (NPs) have been demonstrated to serve as extremely versatile theranostic nanovectors since they act as an imaging probe and as the therapeutic agent for hyperthermia at the same time [25–27]. Another successful approach involves the use of magnetic nanoparticles (MNPs) [28–30] or the combination of MNPs (acting as contrast agents) with mesoporous silica nanoparticles with high surface areas to maximize the drug loading efficiency of the overall theranostic system and allow for a stimuli-responsive release of the therapeutic payload [31,32]. Other techniques currently being investigated involve photothermal, photodynamic or sonodynamic therapy in combination with other imaging tools such as optical imaging, magnetic resonance imaging and ultrasound imaging [33–36].

Nanomaterials have also been successfully applied in tissue engineering (TE) as tools able to promote the repair of damaged tissues and organs [37]. Similar to the other nanomedicine applications previously mentioned, the advantage of using nanoparticles in TE derives from their small size and large surface to volume ratio. Among others, these features allow for an easy surface functionalization of the particles with peptides, ligands or proteins, their easy diffusion across membranes and facilitated cellular uptake. Moreover, nanoparticles can show superior biocompatible properties in some cases, while the nanoparticles’ size resembles the nanometric scale of components present in the extracellular matrix of tissues. Carbon-based nanomaterials, as well as metal oxide- and silica-based nanoparticles, have been used either alone or else in combination with other polymeric materials for the fabrication of smart composite scaffolds with improved functionalities in terms of their biological, electrical and mechanical properties [38–41]. The use of metal/metal oxide nanoparticles also led to very promising antibacterial activity, thus regulating bacterial infections often present after surgery to remove/replace organs and tissues [42]. In other cases, the use of ceramic and metal oxide nanoparticles allowed for the induction of the remotely-controlled mechanotransduction of cells, with evident effects on cells proliferation and tissue regeneration rates [43,44]. The use of magnetic nanoparticles for remote cell patterning was also successfully demonstrated [45].

As expected, a plethora of nanomaterials, both of organic and inorganic derivation, have been successfully explored as smart functional materials in nanomedicine. In the specific case of ceramic materials and metal oxides, some examples include: mesoporous silica nanoparticles as nanocarriers in drug delivery systems [46,47] and as bioactive agents for bone tissue engineering [48]; cerium oxide nanoparticles featuring antibacterial and antioxidant therapeutic properties [49–51]; magnetic iron oxide nanoparticles, which were successfully proposed as theranostic nanovectors coupling therapeutic effects against cancer cells through hyperthermia with MRI contrast agent properties [52–54].

More recently, the use of zinc oxide (ZnO) in nanomedicine captured considerable attention [55]. ZnO is a low-cost, abundant material, classified as a generally recognized as safe (GRAS) material from the Food and Drug Administration [56], which makes it suitable for a plethora of biomedical applications. Moreover, ZnO is a wide band-gap semiconductor (3.37 eV) featuring peculiar physical and chemical properties, such as photo- and sono-catalytic activities [57–59], piezoelectricity [60,61] and pyroelectric [62] behaviors. The abovementioned properties can be combined together and even strengthened by selecting, among the numerous ZnO morphologies, the most appropriate one: thin films [63,64], nanowires (NWs) [65], nanorods (NRs) [66], nanobelts [67], nanoparticles (NPs) [68] and flower-like structures [69] can be easily synthesized by wet and dry preparation approaches such as sol-gel and hydrothermal routes, and vapor-phase deposition techniques.

Various ZnO nanomaterials have been successfully applied in TE [70]. ZnO NWs [71], NRs [72] and nanoflowers [73] have been found to promote the adhesion, growth, and differentiation of several cell lines. ZnO NPs can be easily addressed in the human body due to their reduced size, which also facilitates cellular uptake [74]. Therefore, the promising osteogenic and angiogenic properties of ZnO NPs have been demonstrated, along with their use as nanotherapeutic agents to fight bacterial infections [75,76]. In this sense, ZnO NPs are defined as nanoantibiotics due to their promising antimicrobial properties against both Gram-positive and Gram-negative bacteria.

ZnO nanomaterials as targeted drug delivery systems have been reported as well [77–79]. ZnO QDs (2–10 nm) were combined with doxorubicin-loaded Mesoporous Silica Nanoparticles (MSNs) and used as nanolids to cover silica nanopores and achieve a sustained and pH-triggered delivery of the anticancer drug [80]. Other works include the use of mesoporous ZnO nanospheres [81], dandelion-like mesoporous ZnO capsules [82] and porous hexagonal ZnO nanodisc structures [83]. In the latter cases, the targeted and pH-triggered delivery of chemotherapeutic agents against cancer cells was effectively demonstrated. ZnO nanoparticles have been also used as imaging agents [79] for cell labeling [84], tumor targeting [85] and diagnostics [86]. ZnO NPs can also serve as a nanodrug to treat cancer cells [68,87,88]. The therapeutic effect is accomplished by the release of harmful species (Zn^2+^ cations and reactive oxygen species, ROS) upon interaction of ZnO with aqueous media [89–91]. The therapeutic efficacy of ZnO NPs against different cancer cell lines was confirmed in vitro. In some cases, the remote activation of ZnO NPs cytotoxicity has also been obtained. It is the case of photodynamic [92] and sonodynamic [89,93] therapeutic approaches, an external stimulus (a light or mechanical one) was able to activate the cytotoxic effect of the nanotherapeutic drug, i.e., the ZnO NPs themselves, which could generate a harmful ROS or mechanical damage to cancer cells.

Despite the promising results, the use of ZnO nanomaterials in nanomedicine is still limited because of some intrinsic limitations such as a low stability of the ZnO particles in biological fluids and the non-controllable release of Zn^2+^ cytotoxic species [94,95]. Hence, additional efforts are required to make ZnO more attractive. For this purpose, the doping of ZnO served as a powerful approach to confer on ZnO new functionalities rather than to improve pre-existing ones. The various physical and chemical properties of ZnO NPs have been properly customized for a specific function by inserting *ad-hoc* chemical elements (dopants) into the ZnO crystalline structure. For example, the optical [96], electrical [97], electromechanical [98] and catalytic [58] properties could be optimized and strengthened after doping ZnO with appropriate elements. In other cases, doped ZnO nanomaterials showed unprecedented properties with respect to the pure material counterpart [99]. Therefore, ZnO doping represents an intriguing solution to overcome some of the abovementioned limitations and, to a further extent, a way to use ZnO NPs in nanomedicine.

The interest in using doped ZnO nanomaterials for nanomedicine is quite recent and the literature about this topic is growing rapidly. Even though the potentiality of pure ZnO nanomaterials applied to biomedicine has been already highlighted by other works, to the best of our knowledge no literature reviews have stressed the important progress and manifold biomedical applications that can be achieved by using doped ZnO nanoparticles. Therefore, this review covers the more recent achievements in the use of doped ZnO NPs for applications in nanomedicine. The first section summarizes the most diffuse elements for doping ZnO nanoparticles. Then, a general overview of the main synthetic approaches used to prepare doped ZnO NPs is provided. Finally, the main applications of doped ZnO NPs in nanomedicine are reviewed, with a particular focus on their use as antimicrobial agents, contrast agents for bio imaging applications and as therapeutics against cancer.

## 2 Materials for Doping ZnO Nanoparticles (NPs)

Zinc oxide (ZnO) is an inorganic material that has been successfully applied in numerous fields, ranging from gas sensing [100] and energy harvesting [101–103] to biomedicine [70,104], to name only a few. In addition to the properties mentioned above, it may be useful to add further functionalities to ZnO rather than to improve the pre-existing ones, with the final aim to design a multifunctional system.

**Figure 1:**
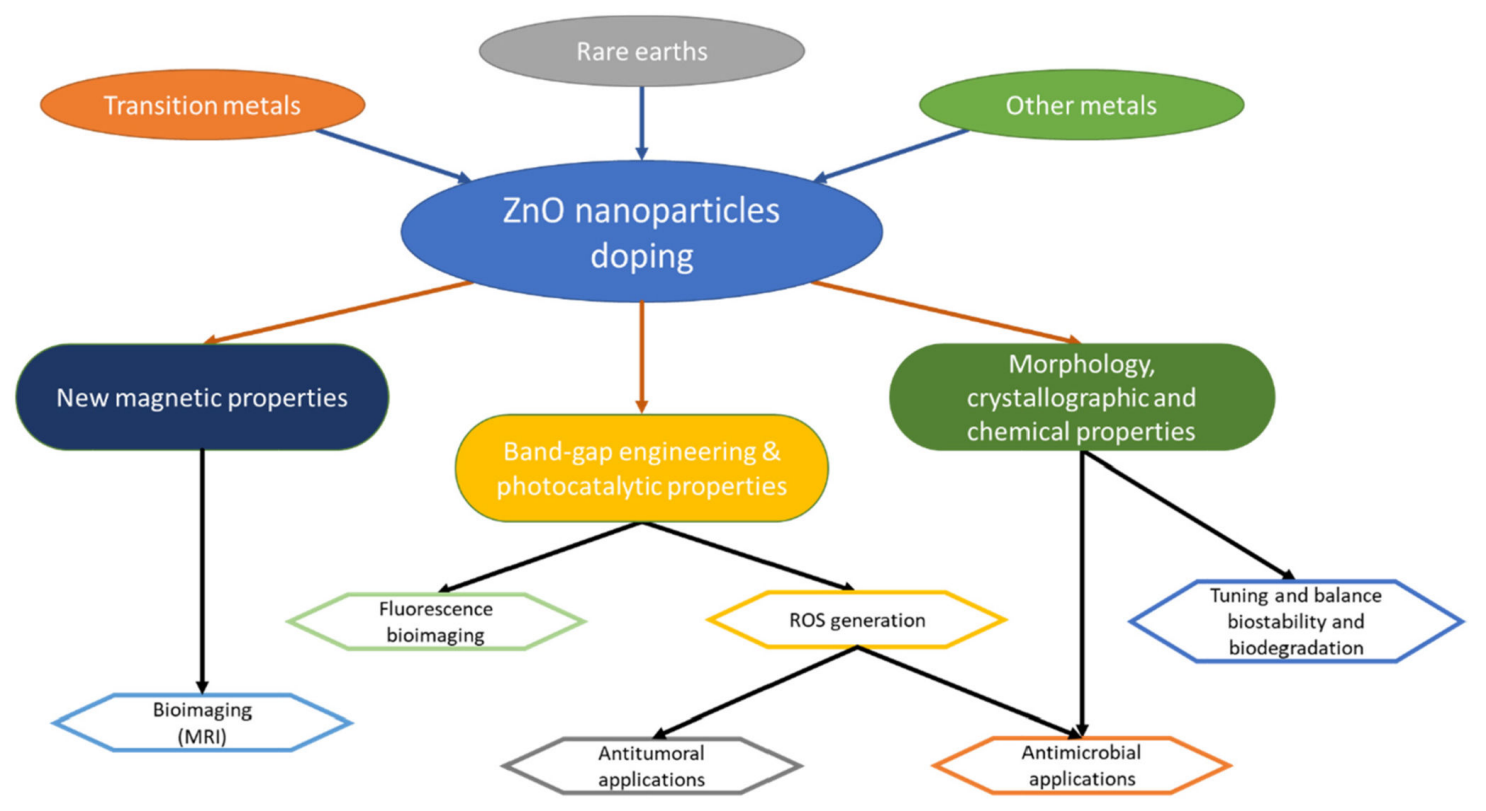
Main applications of doped ZnO nanoparticles in nanomedicine.

For this purpose, doping represents a valuable approach to tune various ZnO properties. Doping consists of the insertion of a specific ion into a crystal lattice, not originally present in the starting material. It can be particularly useful to modulate the energy band-gap, with a direct consequence on the ZnO photocatalytic properties and related antimicrobial activity. Moreover, the insertion of selected elements in the ZnO lattice allows for the generation of a weak ferromagnetic behavior in the doped particle [105–107], the tuning of the degradation properties in aqueous environments [58,108], or even the modulation of the electromechanical response [98,99]. All these aspects, summarized schematically in Figure 1, may be exploited in nanomedicine to design a multifunctional theranostic nanoplatform able to perform both diagnoses (for example, through magnetic resonance imaging) and therapy (such as photodynamic therapy or enhanced reactive oxygen species generation) at the same time.

The physical and chemical properties of doped ZnO are strongly dependent on the doping element. Actually, several aspects, such as the ions’ dimensions, the electronegativity, the coordination state etc., all contribute to determine the final properties of the doped material [109]. For these reasons, the use of *ad-hoc* dopants must be foreseen on the basis of the expected new functionality. In the following, the most used ions reported in literature are briefly presented, and their effects, due to their inclusion in the wurtzite ZnO crystal lattice, are summarized.

### 2.1 Rare Earth (RE) Elements

Rare earth (RE), or lanthanide, elements are widely exploited for the doping ZnO nanomate rials and to properly modify the corresponding electronic band structure [110]. As highlighted in the work of C errato et al. [111], up on UV light irradiation, ZnO presents a very fast recom bination of the photogenerated charge carriers. This results in a low quantum efficiency when this system is used as a photocatalyst. The use of lanthanide, due to the 4f configuration, may help in increasing the recombination time of the electron/hole pair in the semiconductor [111], resulting in a higher efficiency of the system in the photocatalytic process, with consequences on the antimicrobial activity. Cerium, for exampte, lias been proven to enhance the photocatalytic properties ot ZnO nanorods through this mechanism [112], exceeding the performance of titanium dioxide (TiO_2_), which is the material typically exploited as a reference for these purposes.

**Figure 2:**
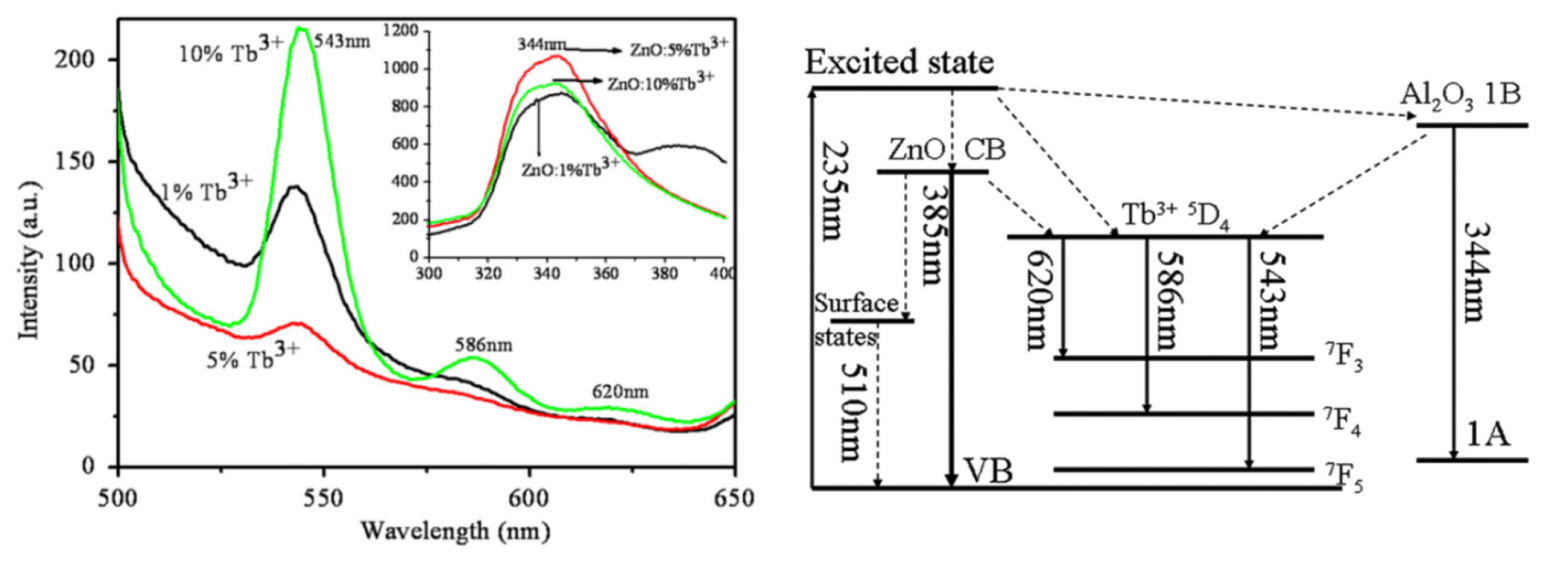
Photoluminescence properties of Tb-doped ZnO nanotubes. (**a**) Emission spectra of ZnO with different dopa nt levels excited by 235 nm radiation; (**b**) energy-levels schematic with the electron transition processes in Tb-doped ZnO nanotubes grown onto alumina. Adapted from [114].

The change in the band-gap and the generation of trap states make RE doping interesting; also, from the luminescence point-of-view, this increases the possibility oi ZnO being used as an imaging agent. The trap states induced by doping may act as radiati ve recombination sites not present in the pure crystal and the dopant atoms and, together with aspecVs such as the crystallite size, contribute to change the band-gap of the system, resulting in the possibility to tune the optical properties of ZnO. In the work of Kumar et al.[113],it is pointed out that ZnO may have improved luminescence properties front RE doping because of the transitionu with the 4f orbitals of the RE elements in the crystal lattice. For example, Zhao et al., [114] synthes ized an array of terbium-doped ZnO nanotubes which showed three emission bands in the range of visible light (543 nm, V86 nm and 620 nm, as clearly visible in Figure 2a) due to terbium doping. In particular, it was also demonstrated that the 586 nm Vand was due to tire energy transfer between ZnO and Tb^3+^ ions obtained through the electron transitions depicted in Figure 2b.

Doping with RE materials is also a valuable method to change the piezoelectric behavior of ZnO. Indeed, the introduction of defects into the crystal further enhances the asymmetry of the wurtzite crystalline structure responsible for ZnO piezoelectricity. For example the generation of a ferroelectric behavior in lanthanum-doped ZnO was reported, alongside an increase of the piezoelectric coeificient d33 by one order of magnitude (≈101.30 pm/V for La-doped ZnO (mean valuei vs. the accepted value ot 12.4 pm/V for bulk ZnO [115]). The reason for this behavior was attributed to the large ionic radius oi the dopant element (1.22 Å) with respect to zinc’s radius (0.74 Å), which induced a stronger distortion of the crystal cell and the rise of a stronger spontaneous polarization [116].

It is worth noting that, despite transition metals dopants being the best choice to induce room temperature fRT) ferromagnetism in ZnO, gadolinium, an RE element, has been proven to successfully induce a magnetic behavior in ZnO nanoparticles [117,118]. In particular, RT ferromagnetism was observed for a film made of Gd:ZnO nanop articles, doped by low energy fon implantation method. The authors iound that a thermal annealing at 650 °C was able to favor the diffusion of the; dopant atoms into the ZnO lattice. The as-prepared ZnO:Gd film showed a RT ferromagnetism and the formation of some ruper-paramagnetic clusters [119]. The reason of this behavior is still under debate and the use of RE doping elements in general, and gadolinium in particular, is to induce magnetism in ZnO nanoparticles, which is reported by tew works. On the contrary, most of the research on magnetic ZnO nanoparticles is focused on the use of transition metals, ar disc ussed in the next section.

In conclusion, RE elements have been more extensively studied to increase the photocatalytic and optical properties of ZnO nanomaterials, but also to confer on ZnO magnetism and improved electromechanical properties in some cases.

### 2.2 Transition Metals (TM) Elements

Another class of elements that have been widely exploited as doping agents for ZnO are the transition metals. Elements such as cobalt (Co) [120], chromium (Cr) [121], iron (Fe) [122], manganese (Mn) [123] and copper (Cu) [124] have all been successfully used to enhance pre-existing properties or to give new ieatures to ZnO, moking it interesting also from the nanomeclicine point of view.

The research on TM-doped ZnO gained lots of attention because of the ability of th is material to work as a diluted magnetic semiconductor (DMS). DMSs are semiconducting materials that also present a ferromagnetic behavior because of the presence of transition metal (TM) ions that are ferromagnetic in their pure form, such as Mn and Fe. Despite such behaviors being still under debate [125], numerous works have reported the observati on of RT ferromagnetism in TM-doped ZnO nanomaterials.

As in the case of RE elements, dopmg with TMs does not only contribute to tire ferromagnetic behavior of ZnO—both the optical [126] and electrical [127] properties cfn be tuned by these materials and are heavily a ffected by several parameters such as tìae oxidation state, ionic radius, electronegativity and olher features oi the doping ion [109].

Among TM dopants, iron (s one of the most used. This element can exist into two different oxidation states (Fe^2+^ and Fe^3^). They both induce a different effect on both the electrical and structural properties, due to the different ionic radius and charge carried in the system. The studies on Fe-doped ZnO are mainly focused on the analysis of the corresponding magnetic properties. However, some works also showed that Fe doping represents an effective approach to improve the chemical stability of ZnO nanoparticles in aqueous media [128] or to enhance the electromechanical response [129]. As an example of such a a study, in the work of Srinivasulu et al. [130], an increasing ferromagnetic behavior at room temperature is reported with the increase of iron doping in ZnO thin films. Additionally, another work shows that the ferromagnetic behavior of Fe-doped polycrystalline ZnO powders is influenced by the post-synthesis annealing conditions [131]. In particular, the ferromagnetic response was optimized by means of an annealing in a hydrogen atmosphere, while an argon atmosphere annealing would not produce any ferromagnetic behavior. Still, the reason for the rising of such a behavior is not clear and controversial explanations are currently reported in the literature. In terms of the piezoelectric properties of Fe-doped ZnO, the work of Luo et al. [129] focuses the attention on the chemical state of Fe ions introduced in the ZnO crystal. According to this work, the oxidation state of the dopant ion plays a fundamental role in determining the electromechanical response of the doped ZnO films because of the different ionic radius; a smaller ion (Fe^3+^) leads to a higher piezoelectric coefficient (~128 pC/N vs. 9 pC/N for 1.2 at.% Fe doping) than doping with a larger one (Fe^2+^).

Manganese has been also extensively used as a dopant agent. Mn is characterized by several oxidation states with a higher difficulty in controlling this parameter. In addition, in this case, the dopant agent is used to modify the optical, structural and magnetic properties of the resulting ZnO semiconductor [118]. The ferromagnetic behavior of Mn-doped ZnO was found to be dependent on the doping amount but without a linear relationship between the two parameters [123]. Another work pointed out that the behavior of Mn-doped ZnO was diamagnetic at 1 at.% of doping and ferromagnetic at 5 at.% [126]. Additionally, the piezoelectric response of Mn-doped ZnO nanoparticles was found to be heavily dependent on the amount of Mn ions introduced in the ZnO crystal but not in a linear way. In the work of Pan et al. [109], the d_33_ piezoelectric coefficient is found to first decrease with the increase of Mn doping up to 4.8 at.% and then it increased with further Mn doping. This behavior is attributed to the change in the oxidation state of Mn ions because of the use of different doping amounts. A different Mn oxidation state means a different ionic radius and hence, a change in the resistance against the rotation of the oxygen–metal bonds in the ZnO lattice.

Among the TM dopants, the use of copper is reported as a tool to increase the antimicrobial activity of ZnO. Hassan et al. successfully demonstrated the fabrication of Cu-doped ZnO coatings with enhanced antimicrobial activity against *E*. *coli* under light illumination [124]. The higher performance obtained by Cu doping was attributed to an enhanced reactive oxygen species (ROS) generation and the release of cytotoxic zinc and copper ions.

Other works also showed the use of chromium and cobalt ions as doping agents for ZnO. Chromium-doped ZnO nanoparticle films were prepared by reactive magnetron co-sputtering. The incorporation of Cr ions within the ZnO crystal lattice resulted into the rise of ferroelectric properties and it notably improved the piezoelectric response with respect to the pure ZnO films [121]. This induced ferroelectric behavior was attributed to the substitution of some of the Zn^2+^ ions by smaller Cr^3+^ ions, which generates a permanent electric dipole. On the other side, cobalt doping was found to increase the band-gap of ZnO, making it transparent to most of the visible light. Additionally, for this doping element, a room temperature ferromagnetic behavior could be observed in Co-doped ZnO nanoparticles [132].

Noble metals, such as gold and silver, were used in different applications as well. For example, the photoluminescence properties of Au-doped ZnO nanoparticles were investigated [133]. The results showed the presence of additional weak luminescence peaks in the visible spectral range the intensity of which was dependent on the doping amount. Gold and silver doping were also investigated as potential antimicrobial boosters for ZnO [134]. In this work, both Ag and Au ions were found to be suitable for increasing the photocatalytic properties of ZnO. However, Au was not able to improve in a significant way the antibacterial activity of ZnO, while Ag-doped ZnO nanoparticles showed enhanced antifungal activity. In another work, the antibacterial activity of Ag-doped ZnO nanoflowers, obtained by means of a green combustion method, was evaluated. The results highlighted a good antimicrobial activity against both Gram-positive and Gram-negative bacteria, as well as an antifungal efficacy against different plant pathogenic fungal strains [135].

In summary, many TMs have been studied for doping ZnO mainly because of their ability to give ferromagnetic properties to ZnO but also to improve its electrical and optical behavior. In the particular case of nanomedicine, doping ZnO with TMs could represent a valuable tool for the design of new multifunctional ZnO nanomaterials with newly acquired bioimaging features, i.e., magnetic resonance imaging.

### 2.3 Other Elements

Apart from RE and TM elements, other elements have also been exploited as dopant atoms. For example, aluminium has been used mainly for the fabrication of flexible detectors. For example, a stretchable NO_2_ gas sensor was developed by allowing the diffusion of Al ions in an array of vertically aligned ZnO nanorods [136]. A flexible pH sensor based on Al-doped ZnO nanosheets was also obtained. Due to Al doping, the sensitivity of the device was highly improved (50.2 mV/pH) with respect to the pure ZnO nanosheets (34.13 mV/pH) [137].

Magnesium is another common choice for ZnO doping and it was used mainly for improving the optical and electromechanical properties with respect to pure ZnO. As in the case of gold, Mg doping was found to increase the photoluminescence properties of ZnO in the visible spectrum of light [138]. In the same work, the photocatalytic degradation of rhodamine B was proven to be enhanced by Mg doping. Moreover, an enhanced antibacterial activity was also obtained, which was correlated to a lower band-gap and the resulting enhanced photocatalytic activity with respect to pure ZnO. From the electromechanical point of view, in a work from Chen et al., the piezoelectric coefficient of Mg_x_Zn_(1-x)_O appeared to be increased (d_33_ = 54.1 pm/V) by increasing the amount of Mg up to x = 0.28 [139]. After this point, which was found to be the limit for not observing the formation of any secondary phase in the crystalline structure of ZnO, the authors found that the d_33_ coefficient abruptly reduced values below the pure ZnO one.

Nitrogen-doped ZnO nanowires, further coated by a thin layer of titania to form a core–shell structure, were also proposed [140]. After the thermal annealing of ZnO nanowires in a nitrogen atmosphere, the nanomaterials showed a higher absorption of UV and visible light than the corresponding air-annealed samples, due to slightly lower values obtained for the band-gap. Ultimately, the one-dimensional ZnO–TiO_2_ core-shell nanostructures were proposed for photoelectrochemical water splitting, showing enhanced efficiencies under solar light illumination.

Actually, the choice in terms of dopants is very wide, as witnessed by a few other works reporting the use of other elements such as chlorine [141], antimony [142], fluorine [143], lithium [144] and many others, each of which changes specific properties of pure ZnO that may be suitable for a specific application.

## 3 Synthesis Methods and Characterization

**Figure 3:**
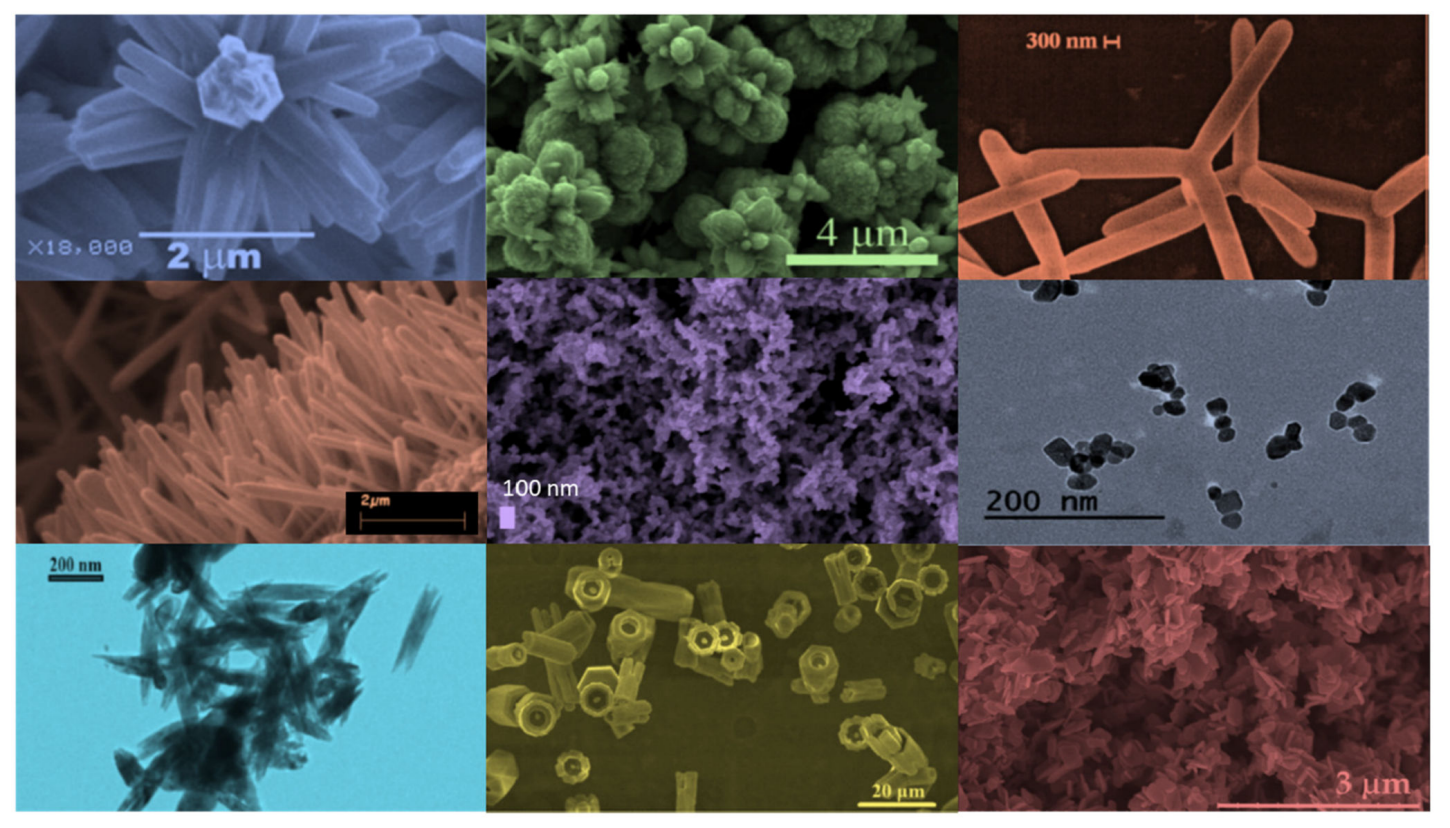
Possible morphologies of ZnO nanostructured systems. From left to right and from top to bottom: nanoflowers (adapted from ref. [149]), nanopods (adapted from ref. [147]), nanorods (adapted from ref. [148]), mesoporous films, spherical nanoparticles, nanoneedles (adapted from ref. [150]), hollow microcolumns (adapted from ref. [151]), and micropods (adapted from ref. [152]).

As already mentioned, one of the main advantages in using ZnO is the availability of a plethora of morphologies which can be easily prepared by various methods [11]. Figure 3 shows the electron microscopy images of microparticles with a very high specific area and various morphologies, such as a flower-like shape [69,145], multipods [146,147], nanorods [148], films with a nanostructured surface [77] and nanoparticles [76] of different shapes and dimensions, all of them well established and reported in the literature. Moreover, the methods through which these particles are obtained are even larger in number than the morphologies themselves. Therefore, it is difficult to make a comprehensive overview of all the possibilities that are already present. By considering the specific subject of this review, the most rapid low-cost and high-yield methods for preparing doped ZnO nanoparticles are discussed.Figure 3 Possible morphologies of ZnO nanostructured systems. From left to right and from top to bottom: nanoflowers (adapted from ref. [149]), nanopods (adapted from ref. [147]), nanorods (adapted from ref. [148]), mesoporous films, spherical nanoparticles, nanoneedles (adapted from ref. [150]), hollow microcolumns (adapted from ref. [151]), and micropods (adapted from ref. [152]).

In the following, wet chemical methods are first presented. This category is probably the most diffuse one, due to the excellent trade-off between the quality of produced materials, versatility and poor requirement in terms of instrumentation, and, in particular, for doping ZnO with rare earth elements. Then, other methods, such as flame spray pyrolysis [128], sputtering [153] or pulsed laser deposition [154], are described as being widely used for the preparation of both doped ZnO films and nanoparticles.

### 3.1 Wet Chemical Methods

Wet chemical methods are among the most promising techniques adopted to synthesize micro- and nanostructured materials. Among the main ad-vantages are the use of non-toxic solvents, low synthesis temperatures, and the use of simple equipment. Moreover, due to the relatively good purity and resulting stoichiometry of the resulting material wet chemical methods appeal to be a promising choice for the development of doped ZnO nanoparticles.

A typical sol-gel/hydrothermal method to prepare ZnO-based materials is based on the following reactions [69]:

(1) $${\text{Zn}}^{2+}+2{\text{OH}}^{-}\to \text{Zn}{\left(\text{OH}\right)}_{2},$$

(2) $$\text{Zn}{\left(\text{OH}\right)}_{2}+2{\text{OH}}^{-}\to \text{Zn}{\left(\text{OH}\right)}^{2-}{}_{4},$$

(3) $$\text{Zn}{\left(\text{OH}\right)}^{\text{2-}}{}_{4}\to \text{ZnO}+{\text{H}}_{2}\text{O}+2{\text{OH}}^{-}$$

Typically, this process is assistedby a mineralizing agent that provides the OH^-^ groups necessary to tune the pH of the solution, avoiding the precipitation of zinc hydroxide aod promoting the formation of ZnO. Typical choices are NaOH [145], KOH [69] and, less olten, NH_4_OH [155].

Among the zinc precursors, the main used are zinc acetate [156,157], zinc nitrate [158,159] and zinc chloride [150,160,161], which readily dissolve in most of the solvents used in this kind of reaction (water [152,162], ethanol [163] and methanol [164]). For the dopant precursor, chlorides [138], nitrates [165,166] and acetates [144] from the corresponding dopant elements are the typical choices.

Then, the inclusion of the dopant can be obtained in a practically straightforward way, by mixing the dopant ion solution together with the zinc precursor solution, before mixing with the base.

Another challenging aspect is the design of particles with an appropriate morphology. Indeed, it play an important role in determining the NPs biological behavior, influencing factors such as cell internalization. The morphology and the dimension of the nanoparticles can be controlled through surfactants such as sodium dodecyl sulphate [167] or cetyltrimethylammonium bromide [168]. Moreover, the use of different surfactants in different amounts has been demonstrated to completely change the final morphology of the particles, leading to very peculiar shapes such as nanoflowers, nanomultipods or even nanopyramids [169].

The sol-gel approach is widely used when doping ZnO with rare earth elements is pursued.

Goel et al. reported the synthesis of Gd-doped ZnO nanorods by means of a wet chemical solution route that involved zinc chloride and gadolinium (III) chloride hydrate dissolved in distilled water [161]. The salt solution was prepared to have Gd^3+^ at a 5 mol.% concentration with respect to ZnO, and it was mixed dropwise to a sodium hydroxide (NaOH) solution in order to get powder precipitation. By TEM and XRD analyses, the authors found useful information about the structural changes occurring in the ZnO wurtzite crystalline structure: after the correct insertion of Gd dopant, there was no other secondary phase in the resulting material. An increase of the unit cell parameters for Gd-doped ZnO (*a* = 3.2524 Å, *c* = 5.2095 Å and V = 47.73 Å^3^) was found with respect to the pure ZnO nanorods (*a* = 3.2518 Å, *c* = 5.2095 Å and V = 47.70 Å^3^), as expected from the larger ionic radius of Gd^3+^ (0.94 Å), with replacement taking place at the Zn^2+^ sites in the host ZnO lattice, which is able to modify the dimensions of the crystal unit cell. This distortion of the crystalline cell leads to an enhancement of the piezoelectric coefficient, which changed from 12.4 pm/V of pure ZnO [115] to 45.49 pm/V for the Gd-doped nanoparticles (maximum value). A change in the unit cell parameters was observed in a large variety of works in which ZnO was doped, as it has been summarized in Table 1.

**Figure 4:**
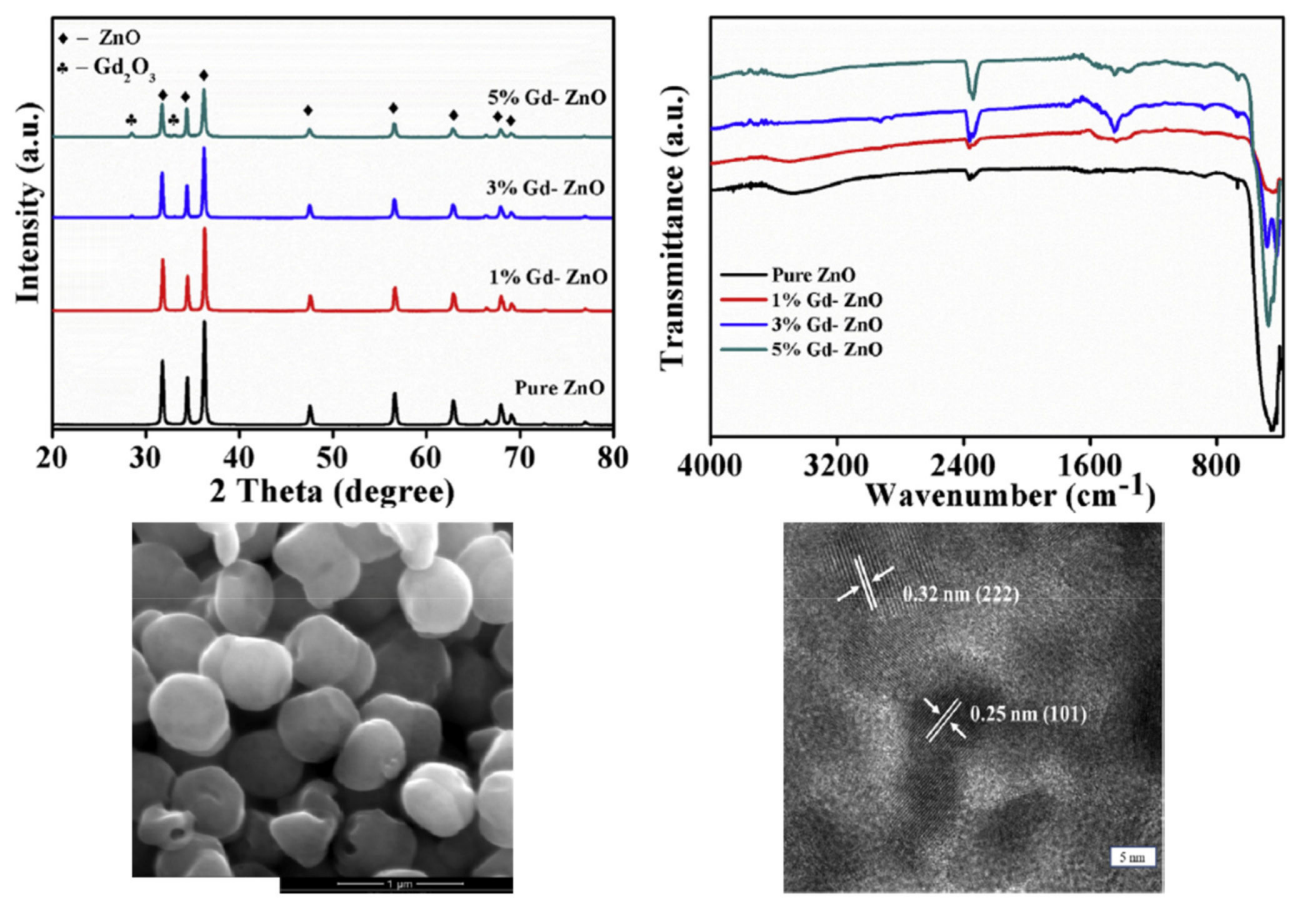
Characterization of gadolinium-doped ZnO nanoparticles. From left to right and top to bottom: XRD patterns and Fourier transform infrared spectroscopy (I^r^TIR) spectra of the nanostructures at different doping levels, scanning electron microscope and transmission electron microscope images of 3% Gd-doped ZnO nanoparticles. Adapted from ref. [177].

In the work of Selvarajet al., gadolinium (III) nitrate hexahydrate was used as a doping precursor as well [177]. Different levels of Gd doping were considered and the photocatalytic properties of Gd-doped ZnO nanoparticles were evaluated. The nanoparticles synthesized in this work, whose electron microscopy images are reported in Figure 4—seen in the bottom panel—were obtained through a co-precipitation method in which the metal precursors were first mixed together at a proper ratio and then NaOH was added to allow the precipitation of the particles. In this case, after the synthesis, the obtained samples were also calcined at 500 °C for 2 h. The XRD pattern of the resulting samples (Figure 4, top left panel) showed that 5 mol.% Gd-doped ZnO nanoparticles presented a secondary phase. Therefore, this level of Gd doping can be assumed as the Gd^3+^ solubility limit for this synthesis. Fourier transform infrared spectroscopy (FTIR) was also carried out (Figure 4, top right panel) and it evidenced that the peak associated with the Zn—O bond (443 cm^-1^) was broader in the doped ZnO nanoparticles, due to the deformation of the lattice. A reduction of the band-gap energy value was also reported for increasing the amount of doping from 0 mol.% up to 3 mol.% of Gd^3+^, while an increase was observed for 5 mol.% of doping. The reduced band-gap energy was ascribed to quantum confinement effects deriving from the reduction of the crystallite size as the Gd percentage increased.

Europium (Eu) doping was also demonstrated as a valuable tool to enhance the X-rays absorption properties of ZnO nanoparticles in cancer radiation therapy. In this case, Eu-doped ZnO nanoparticles were synthesized through a chemical precipitation technology [164]. Zinc acetate was dissoived in methanol togethor with europium (III) nitrate pentahydrate (5 mol.% of Eu doping) and the oxide formation was achieved through an NaOH addition. The resulting 8 fo 9 nm sized ZnO:Eu nanoparticlea showed an increased ability to generate reactive oxygen species, when subjected to X-rays, with respect to pure ZnO nanoparticles.

Other RE elements such as lanthanum (La) and cerium (Ce) were successfully used to prepare doped ZnO nanoparticles by the sol-gel method. For example, zinc nitrate and lanthanum (III) chloride heptahydrate were used to synthetise ZnO:La nanoparticles with improved light absorption properties and were successfully used as photo-anodes in dye-sensitized solar cells [116]. Ce-doped ZnO nanorods were fabricated starting from zinc chloride and cerium chloride [171]. The correct insertion of La and Ce dopants within the host ZnO crystal was confirmed by XRD. In both the cases, the wurtzite diffraction peaks present in the pattern of the doped nanorods were shifted toward higher diffracting angles with respect to the pure counterparts, evidencing a higher crystalline defectiveness induced by the correct inclusion of the ions in the lattice.

Additionally, yttrium (Y, considered both an RE and TM element) was successfully included into the crystal lattice of ZnO nanosheets obtained by a co-precipitation method that involved the use of zinc chloride and yttrium (III) chloride hexahydrate. As reported by Sinha et al., a huge increase of the piezoelectric coefficient d_33_ (up to 420 pm/V against 12.4 pm/V of pure ZnO) in Y-doped nanosheets was measured [160]. The XRD pattern of the Y-doped and undoped ZnO nanosheets revealed a shift toward higher angles of the peaks in the case of the doped material, evidencing a change in the lattice spacing among atoms. This aspect, together with the different charge of the inserted dopant and the changed morphology that the inclusion of the dopant has led, are considered the factors which increase the piezoelectric coefficient.

Differently from RE elements, for which wet chemical methods are the most frequently used to develop doped ZnO nanoparticles, transition metals have a plethora of synthesis methods available. However, sol-gel and co-precipitation methods are still the most used in the literature.

Among TMs, iron has been extensively studied as a dopant for ZnO due to its ability to induce a ferromagnetic behavior to the system [178–180]. Many works have dealt with flame spray pyrolysis synthesis [122,128,181], but a lot of works have also reported a wet chemical method to obtain Fe-doped ZnO nanoparticles. Samanta et al. reported the synthesis of Fe-doped ZnO nanoparticles (Fe_x_Zn(_1-x_)O) with dimensions below 50 nm through a sol-gel method [155]. Zinc acetate dihydrate and ferric nitrate were dispersed in distilled water together with citric acid and ethylene glycol as anti-agglomerating agents at a temperature of 65 °C. Aqueous ammonia was added to precipitate the powders that were successively calcined. No secondary iron oxide phases were observed up to x = 0.15 of iron doping. Moreover, the unit cell volume of the ZnO:Fe nanoparticles crystal slightly changed with respect to the pure ZnO counterpart, with a not-proportional trend with respect to the dopant amount. This was attributed to the different fractional substitution of the two alternative coordination numbers of iron (Fe^2+^ and Fe^3+^) and their respective different bond lengths with oxygen. A general increase of the band-gap energy value was also found but without a direct proportionality with the amount of doping. In particular, the maximum increase was obtained for the x = 0.06 Fe ratio. In another work, the attention was focused on the antibacterial activity of Fe-doped ZnO nanoparticles obtained by a sol-gel method. Zinc nitrate hexahydrate, iron nitrate nonahydrate and gelatin were dissolved in distilled water and the resulting nanoparticles showed a dimension of approximately 20-30 nm [182]. This study reported a reduced 2θ angle of the (101) diffraction peaks together with a general decrease of the intensity of the wurtzite diffraction peaks by increasing the amount of Fe doping, which is attributed to an increased disorder of the ZnO lattice after Fe doping.

Manganese-doped ZnO nanoparticles were successfully prepared by a wet chemical method as well. The electronic and magnetic properties of the as-prepared Mn-doped ZnO nanoparticles were investigated [183], by changing the amount of the Mn dopant between 0.5 at.% and 3 at.%. The particles were synthesized from zinc nitrate and manganese nitrate being dissolved in ethanol and polyethylene glycol (PEG). Both the XRD patterns and TEM images showed that Mn doping sensibly reduced the particle size to 20-50 nm.

Cobalt is another commonly used TM element exploited as a dopant ion for ZnO. The sol-gel method was successfully used also in this case, as shown by the work of Lima et al. In this study, the promising photocatalytic and antibacterial properties of Co-doped ZnO were reported [184]. Zinc nitrate and cobalt nitrate were dispersed in aqueous diluted polyvinyl alcohol (PVA) and mixed together. The solvent was dried without the use of any mineralizing agent up until the gel formation, which was finally dried and calcined to form the nanoparticles. Cobalt was successfully inserted in the ZnO crystal without the formation of any secondary phase (up to 10 mol.%) of doping, and an increase of the unit cell volume was observed by increasing the amount of doping. The particles showed an average size below 40 nm and revealed a red shift of the UV-visible photo-absorption spectrum together with a reduction of the band-gap energy. The changes in the optical properties of ZnO:Co nanoparticles were attributed to the increase of the unit cell volume (due to the quantum confinement effect) and to the introduction of trap states in the band-gap, in agreement with other works such as the one by Kayani et al. [132]. On the contrary, a different behavior was found by Manjula et al. [185]. In this case, the nanoparticles were produced again by a co-precipitation method involving zinc acetate and cobalt acetate, and a blue shift of band-gap was observed with an increasing amount of dopant. These contrasting results evidence that a complete understanding of the mechanism of Co doping ZnO nanoparticles has not been reached and many other aspects have to be considered. Moreover, it is worth mentioning that a reduction of the photocatalytic activity was observed by other works [184,185]. This suggests that additional efforts are required to better understand the role played by Co dopants in determining the physical properties of the resulting ZnO nanoparticles.

**Figure 5:**
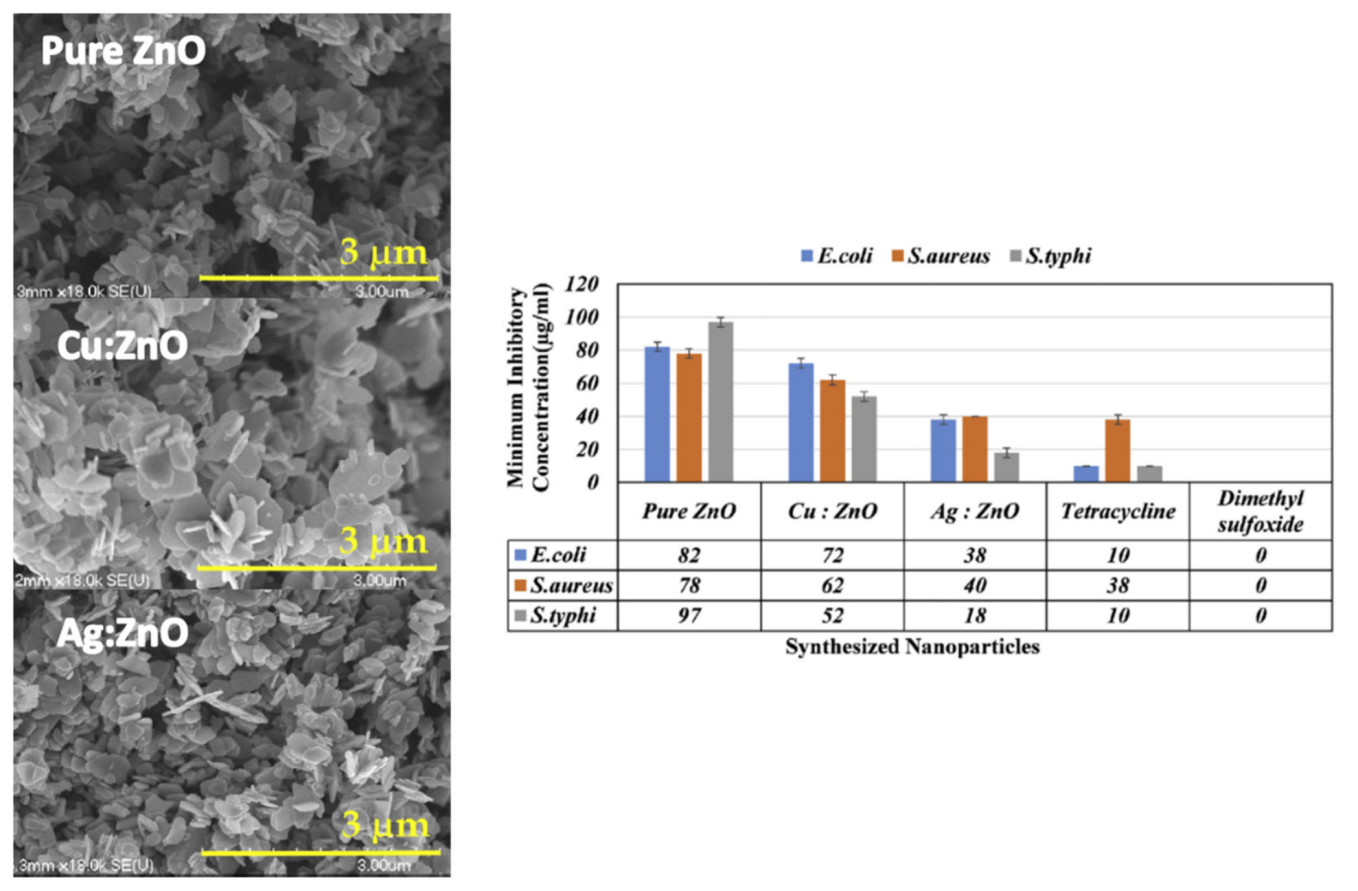
SEM images and antimicrobial performances of Cu- and Ag-doped nanoplates Adapted from ref. [152].

Wet chemical routes were also used to prepare copper-doped ZnO nanoparticles. An example is represented by the work of Abinaya et al. [152], where 5 mol.% Cu-doped ZnO nanoplates were synthesized by a hydrothermal method. Briefly, zinc acetate and copper (II) acetate monohydrate were dissolved in water, and NaOI I aqueous solution was then added to the· solution until a gel formed. Differently from the co-precipitation and sol-gel techniques previously discussed, in this case the gelled solution was placed in an autoclave at 160 °C for 5 h to get the formation of the ZnO:Cu powders. The as-prepare d Cu-doped nanopiates exhibited variable dimensions of few hundreds of nm, as shown in Idgs?re 0. Additionaliy, in this case, the dopant agent slightly increased the unit cell votume ot the ZnO crystal. Cu-doped nanoplates were fested in terms of thetr antimicrobial properties against *E*. *coli* and *S*. *aursus,* showing a decrease in the minimum inhibitory concentration (MIC) with respect to pure ZnO (Figure 5).

Silver is a noble metal widely explored for antimicrobial applications due to the action of Ag^+^ on bacterial membranes [186]. Therefore, it was considered as a TM element to prepare antimicrobial Ag-doped ZnO nanoparticles. A study going in this direction is reported by Sharma et al., in which zinc acetate and silver nitrate were chosen as precursors, while ammonia was used as agent for inducing particle precipitation [165]. In addition, in this case, Ag doping induced a shift in the diffraction pattern toward higher angles with the increase of the doping amount.

Other metals such as magnesium, lithium and aluminium were used for doping ZnO nanoparticles by wet chemical synthesis. For magnesium, the enhanced photocatalytic activity of Mg-doped ZnO nanoparticles with respect to their pure counterparts was reported [138]. The doped nanoparticles were prepared by a co-precipitation method and the effective insertion of an Mg dopant in the host ZnO crystal lattice was confirmed by XRD which highlighted an increase of the volume of the unit cell.

With regard to lithium doping, a work reported its use in enhancing the photoactivated ROS generation of ZnO when using this system for anticancer therapy [144]. In this work, zinc acetate dihydrate and lithium acetate dihydrate were used as precursors in a polyol synthesis performed in triethyleneglycol (TREG) as the solvent.

Finally, Al-doped ZnO (AZO) nanoparticles were synthesized by a wet chemical method and their antibacterial performances were studied. Zinc nitrate hexahydrate was mixed with the aluminium precursor (aluminium nitrate nonahydrate) for the oxide synthesis [187].

Among wet chemical syntheses, the reverse-micelles microemulsion method was also explored for the preparation of doped ZnO nanoparticles [188]. The presence of specific surfactants by this approach can lead to peculiar ZnO morphologies, such as hollow structures [189], and can also limit the dimensions of the particles during nucleation. For example, the reverse-micelles method has been successfully exploited to develop Eu-doped [190,191] and Mn-doped [192] ZnO nanorods and nanoparticles with a controlled size. In particular, in the case of europium-doped nanoparticles, it is possible to obtain either very small nanospheres with an average diameter of about 5 nm, or nanorods with lengths of some hundreds of nanometers and a diameter of some tens of nanometers, by changing precursors (Zn(CH_3_COO)_2_·2H_2_O and Eu(CH_3_COO)_3_·4H_2_O for nanospheres and ZnCl_2_ and EuCl_2_ for nanorods), oils and surfactants (sodium bis(2-ethylhexyl) sulfosuccinate and heptane for nanospheres and octane, CTAB (cetyltrimethyl ammonium bromide) and butanol for nanorods).

As a summary, it may be stated that wet chemical methods are the mostly exploited ones for the development of doped ZnO nanoparticles. The main advantages of these techniques are the simplicity of the process and the wide variety of morphologies obtainable. Table 2 summarizes the main aspects of some of the techniques used to synthesize doped ZnO nanoparticles.

### 3.2 Combustion Methods

Apart from wet chemical methods, combustion-based synthesis techniques have been also explored as an alternative for the synthesis of doped ZnO nanoparticles.

In solution combustion methods, the precursors of the desired material, i.e., zinc and dopant element precursors, are firstly dissolved in a fuel (urea, glycine fuels or citric acid) [197]. Then, the solution is placed in a muffle furnace pre-heated to a temperature higher than the ignition temperature of the fuel. During the combustion of the fuel, a strong exothermic reaction takes place between the fuels and the oxidizing agents present in the solution, generating the target oxide and gaseous species [197].

This gas generation leads to an expansion of the volume of the solid phase and a rapid decrease of its temperature, leading to the formation of ultra-fine and well dispersed powders [197]. This last aspect is very important in biomedical applications where the formation of clusters must be avoided as much as possible since it affects the cellular uptake of nanoparticles.

By tuning the amount of fuel in the solution, various ZnO nanomaterials were synthesized through this versatile synthesis mechanism, showing different morphologies such as pyramid-like particles, nanodisks [198] or nanoparticles [199].

**Figure 6:**
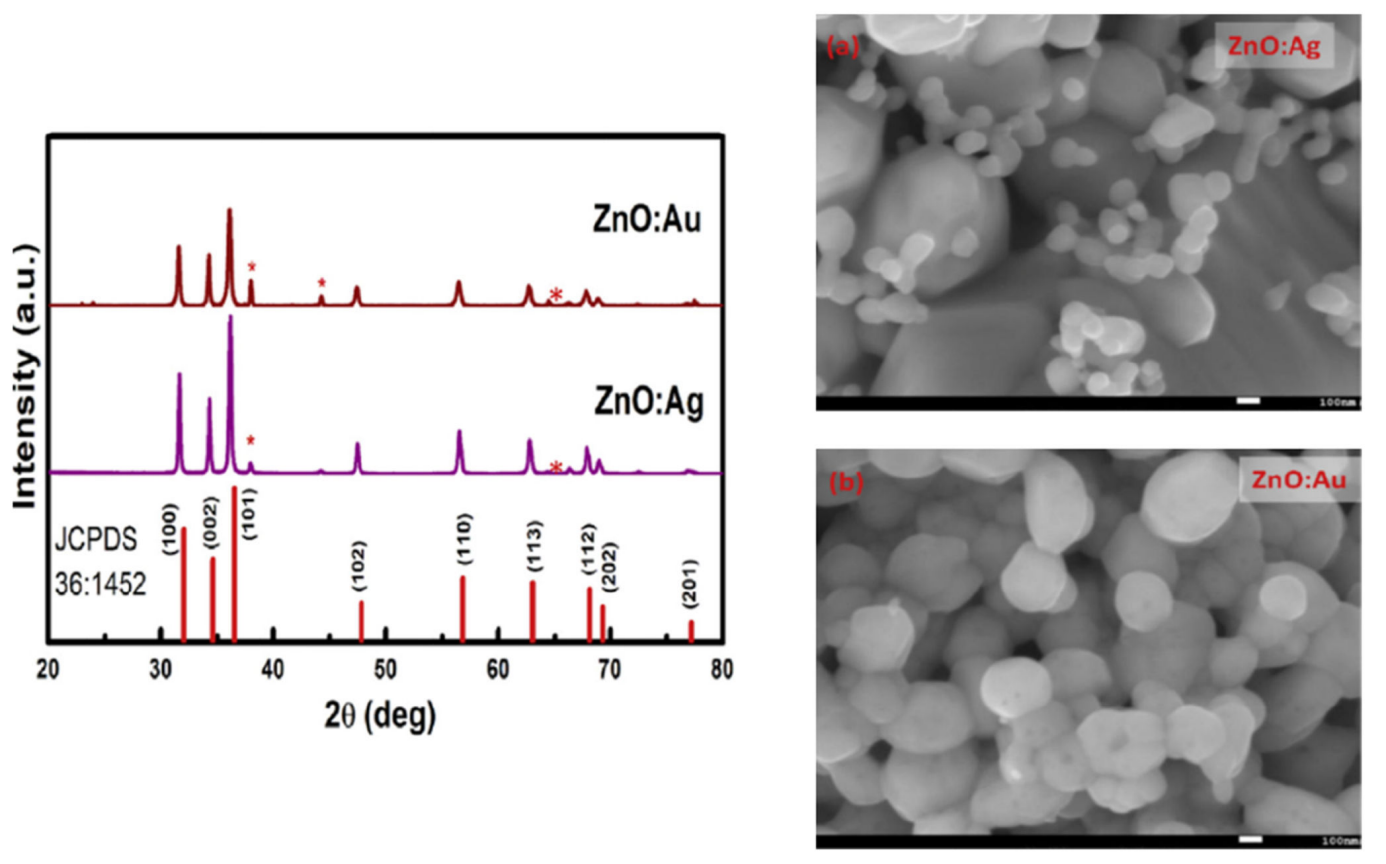
Au- and Ag-doped nanoparticles synthesized through a combustion method. On the left is the XRD patterns of the resulting particles, highlighting the presence of further peaks related to the secondary phases. On the right, the corresponding scanning electron microscope images of Ag-doped (a) and Au-doped (b) nanoparticles. Adapted from ref. [134].

Concerning the doping of nanomaterials, different ions have been successfully included into ZnO. An example is represented by noble metals such as gold and silver. In the work of Pathak et al., both Ag- and Au-doped ZnO nanoparticles were synthesized by a solution combustion method [134]. Zinc nitrate hexahydrate was mixed in urea (used as fuel) together with a small amount of silver nitrate or tetrachloroauric-III-acid hydrate to act as dopants. The obtained solution was dissolved in 5 mL of water pre-heated to 80 °C and then placed in a furnace at 500 °C to start the ignition. Differently from sol-gel methods, the dopant agents generated the secondary phases of noble metals, as observed from the SEM images and XRD patterns reported in Figure 6.

ZnO was also doped with nickel by a solution combustion method. In the work of Silambarasan et al., zinc acetate, nickel (II) acetate, ethanol and ethylene glycol were mixed together and inflamed to get the doped ZnO powder [200]. The XRD patterns showed the high crystallinity of the material. However, also in this case, the doped samples showed additional diffraction peaks related to an NiO secondary phase.

A similar problem was observed for vanadium-doped ZnO nanoparticles. In this case, zinc nitrate hexahydrate and glycine were used as a precursor and to fuel for the reaction, respectively [173]. The dopant precursor was ammonium metavandate (NH4VO3) while water was used as solvent. The XRD analysis performed on the burnt powders showed again the presence of additional peaks related to the formation of a secondary phase, especially for high doping levels.Figure 6. Au- and Ag-doped nanoparticles synthesized through a combustion method. On the left is the XRD patterns of the resulting particles, highlighting the presence of further peaks related to the secondary phases. On the right, the corresponding scanning electron microscope images of Ag-doped (**a**) and Au-doped (**b**) nanoparticles. Adapted from ref. [134].

In another work, Ti-doped ZnO powders obtained by a solution combustion technique exploited titanium (IV) isopropoxide as a dopant precursor [201]. In this case, the additional XRD peaks due to TiO2 appeared only at the highest level of doping (7.5 at.%), while Ti ions were correctly inserted in the host ZnO lattice for lower dopant concentrations (up to 5 at.%).

The solution combustion method was also proposed to synthesize iron-doped zinc oxide nanoparticles in a faster way than sol-gel methods [202]. Zinc nitrate, iron nitrate and urea were used as precursors and fuel, respectively. The solution was heated at 400 °C up to the ignition of the solution and until the formation of a very fragile foam that could be reduced to powder form. Interestingly, the XRD pattern representative of these powders did not present any additional peaks due to the secondary phase up to 5% of Fe doping, while weak peaks related to ferrite were detected only for higher doping levels. However, the formation of secondary phases was not considered a drawback at all. Actually, it was responsible for the promising ferromagnetic behavior observed in the mostly doped samples, even if a weaker hysteresis loop was also found for lower doping levels.

The iron doping of ZnO nanoparticles was achieved by flame spray pyrolysis as well. Differently from the standard solution combustion described previously, the precursor solution is sprayed before setting on fire. Again, a fuel is needed to allow the self-sustainability of the flame as in the case of the solution combustion method [203]. However, a nozzle tip is usually exploited to nebulize the solution precursor.

In the development of doped ZnO nanoparticles, iron is the one that had major advantages in terms of the exploitation of this technique. Many studies have exploited flame spray pyrolysis rather than sol-gel methods to prepare Fe-doped ZnO nanoparticles with an improved biostability in aqueous media [181,204,205]. The improved stability was probably due to the rapidity of this synthesis method, which resulted in nanoparticles with superior surface chemistry properties.

Concerning this aspect, the work of Xia et al. highlighted the decreased dissolution of Fe-doped ZnO nanoparticles in biological media [205]. This doping reduced the amount of Zn^2+^ dissolved in the biological medium with positive consequences on the biocompatibility of the nanoparticles system. The doped nanoparticles were synthesized through flame spray pyrolysis. Zinc and iron naphthalene were mixed separately in xylene. The solutions were sprayed, atomizing the precursor and allowing for combustion through the co-delivery of methane and oxygen. The resulting doped particles were smaller with respect to the pure counterparts and the XRD patterns did not exhibit additional peaks apart from the usual wurtzite phase ones.

Other works reported the use of the same synthesis procedure to develop ZnO:Fe nanoparticles with a good reproducibility [181,204]. Each of these works was focused on the dissolution kinetic of the produced particles and their fate in a biological environment. In all the cases, iron doping was demonstrated to be a very interesting tool to limit nanoparticles’ dissolution and to enhance ZnO cytocompatibility.

As a conclusive remark, it can be stated that combustion methods are not able to efficiently incorporate the dopant agent in the ZnO crystalline structure with respect to wet chemical techniques. This can be the explanation for the limited use of solution combustion routes to fabricate doped ZnO nanoparticles with respect to sol-gel or hydrothermal routes. However, it is worth noting that Fe-doped ZnO nanoparticles obtained by flame spray pyrolysis showed superior properties in terms of their stability in biological media.

### 3.3 Other Techniques

Despite their application being mostly limited to the synthesis of ZnO films, other synthesis techniques for doping ZnO are worthy of mention.

An example is chemical vapor deposition (CVD), which is typically used to grow thin films. In the work of Shuang et al., Mn-doped ZnO flower-like structures were successfully grown in a CVD system and the corresponding optical properties were analyzed [206]. In this case, Zn and MnCl_2_ in powder form were used as solid source materials for growing the flower-like structures. The SEM results reported highly oriented structures, but most of the dopant ions accumulated on the outermost surface of the nanostructures. A Fe/Co doping of ZnO nanowires was obtained by the CVD method as well and the resulting nanostructures were characterized by a high degree of crystallinity and an interesting ferromagnetic behavior [207].

Sonochemical syntheses are recently acquiring a lot of interest as well, since the high energy which is freed due to air bubble cavitation can be exploited to locally overcome the limits of pressure and temperature which commonly affect other techniques [208]. A sonochemical wet impregnation method was instead exploited for the fabrication of Ce-doped ZnO nanoparticles for photocatalytic applications [209]. The doped ZnO nanoparticles were fabricated by keeping in sonication a solution of ZnO nanoparticles and ammonium ceric nitrate in distilled water, and then drying the precipitates.

Other examples include the synthesis of ZnO–graphene nanohybrids, where different morphologies could be obtained according to the synthesis solution pH [210]. The sonochemical approach has been successfully exploited also to dope ZnO with both common and exotic doping elements like dysprosium [211], magnesium [212] and praseodymium [213], revealing the high versatility of this method.

An alternative technique for doping is to mix pure ZnO powders with those of the dopant species, and then thermally anneal the mixed powders together at a high temperature. This method was exploited by Ivetic et al. to obtain Mg-doped nanoparticles and to evaluate the increase of the corresponding photocatalytic activity with respect to pure ZnO [214]. Mechanical alloying—which does not use temperature, but simply mixes and ball mills ZnO and the dopant metal powders together—was able to include the metal into the ZnO particles. With this method, Fe was included in the ZnO matrix up to 7.08% without any clear evidence of the formation of iron clusters [174]. A ferromagnetic behavior was also found in this system.

Finally, other techniques are based on the deposition of doped ZnO coatings and thin films such as magnetron sputtering [129], pulsed laser deposition [215] and many others.

In summary, wet chemical methods are surely the most exploited and promising routes to obtain good quality doped ZnO nanoparticles, with a large variety of precursors and parameters that allow an extreme customizability of the resulting material. Other techniques, like combustion methods, revealed to be useful in terms of the rapidity of reaction but the dopant atom inclusion in the crystalline lattice of ZnO is poor with respect to chemical routes, limiting their applications to systems in which the quality of the material is not a key parameter.

## 4 Use of Doped ZnO NPs in the Biomedical Field

Zinc oxide has been part of many applications in the biomedical field. Many of them are related to its antimicrobial activity [216], but other applications also prove the use ZnO as a therapeutic agent against cancer cells [217,218] or as a tool for tissue engineering [70,78]. However, despite pure ZnO showing many interesting properties, it still presents many factors that need to be further optimized for its further exploitation in the biomedical filed in general, and in nanomedicine in particular.

A first important aspect to be considered is its dissolution behavior in aqueous media. Actually, zinc oxide can dissolve in water generating free Zn^2+^ cations. This can more easily occur in presence of high surface area nanoparticles because of their superior surface reactivity [216]. Zn^2+^ cations are successfully exploited to increase the bactericidal activity of ZnO [219]. However, this aspect may also result in an increased cytotoxicity that could prevent its use in the human body.

Despite the high quality of ZnO as an antimicrobial agent, its performance may be even enhanced if the corresponding photocatalytic activity is further optimized. ZnO is a semiconductor able to generate highly cytotoxic/antimicrobial ROS [220]. Upon interaction with electromagnetic radiation, semiconducting ZnO materials are able to promote electrons from the valence band to the conduction band, generating free electrons which are able to migrate up to the outermost ZnO surface and react with the external aqueous environment, leading to the generation of ROS [92,221]. Band-gap engineering of ZnO may be a valuable tool to increase the efficiency of this process, reducing or increasing the energy required to generate free electrons and controlling the efficiency in ROS generation. This ability in generating ROS can be exploited in photodynamic therapy, transforming ZnO nanoparticles into an interesting weapon against cancer.

Finally, ZnO on its own lacks intrinsic imaging properties which are very useful for various biomedical applications, such as in theranostics. Tuning its dimensions and energy band-gaps, the corresponding optical properties can be properly optimized and interesting luminescent properties can be gathered as in the case of ZnO quantum dots [163,222]. Moreover, as already stated, the inclusion of transition metals in the crystal lattice confers on ZnO a magnetic behavior, paving the way to its exploitation as a contrast agent in magnetic resonance imaging [118].

All these aspects can be fulfilled by doping ZnO with specific elements. In the following, the main findings in this sense will be described.

### 4.1 Biological Behavior

One of the main problems related to the use of ZnO in biomedical applications is its dissolution in aqueous/biological media [223]. ZnO is in fact stable at a basic pH, while it can easily dissolve in an acid environment [224]. The pH of a biological medium depends on several parameters and can change over time. Therefore, it is possible to have a certain degree of ZnO dissolution, which can be even enhanced when the dimensions of the nanoparticles under analysis are highly reduced, exposing a large area to the liquid environment [224]. The dissolution leads to the release of zinc cations (Zn^2+^) which have been proven to be harmful for cells, damaging the cellular zinc homeostasis and, consequently, leading to lysosome and mitochondria damage and cell death [223]. Hence, particular attention should be paid to the control of ZnO dissolution, in order to limit cytotoxicity.

For the chemical design of ZnO-based systems with increased zinc cation stabilities in media, specifically used in medical applications, one successful approach is represented by the methodology based on electrochemical equilibria in aqueous solutions [225]. An alternative approach to improve the biocompatibility and zinc cation stability of ZnO nanoparticles is to cover their surface with inorganic [226–228] or organic [140,229,230] coating materials. However, this approach requires additional synthesis steps and a general increase of the nanoparticles’ size, which may represent a limitation for specific applications.

The best situation would be to prepare ZnO nanoparticles that are intrinsically stable in a biological medium, without the addition of any external layer. This task can be addressed once again by including a dopant atom in the crystal lattice of ZnO.

**Figure 7:**
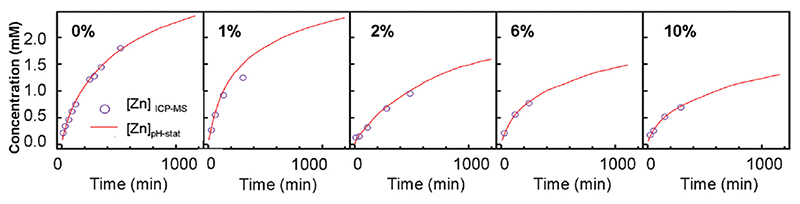
Zinc release in an aqueous solution at pH 7 by the dissolution of Fe-doped ZnO nanoparticles at different doping levels Adaeted from ref. [128].

Actually, Fe doping has been proposed as a stabilizer of ZnO in aqueous media. George et al. [128] demonstrated that the inclusion of Fe ions into ZnO nanoparticles successfully decreased the dissolution rate by measuring the amount of zinc ions released in an electrolytic solution at pH 7 ± 0.04. The Zn^2+^ kinetics were evaluated by measuring the nitric acid amount required to maintain the solution pH at the fixed value. The results showed that, by increasing the amount of doping, the dissolution of the nanoparticles decreased accordingly (Figure 7). This behavior was attributed to the stronger chemical bonds between the iron dopant and the host ZnO lattice, which resulted in a higher difficulty for the Zn^2+^ io ns to be released in the; environment.

First principle calculations confirmed these experimental evidences [108]. The computed energetics evaluated for different iron doping levels showed that iron can stabilize the ZnO nanoparticles. Moreover, in the same work, it was also proven that the coordination state of iron was mainly Fe^2+^ and that the inclusion of the dopant did not damage the crystal lattice in a considerable way.

**Figure 8:**
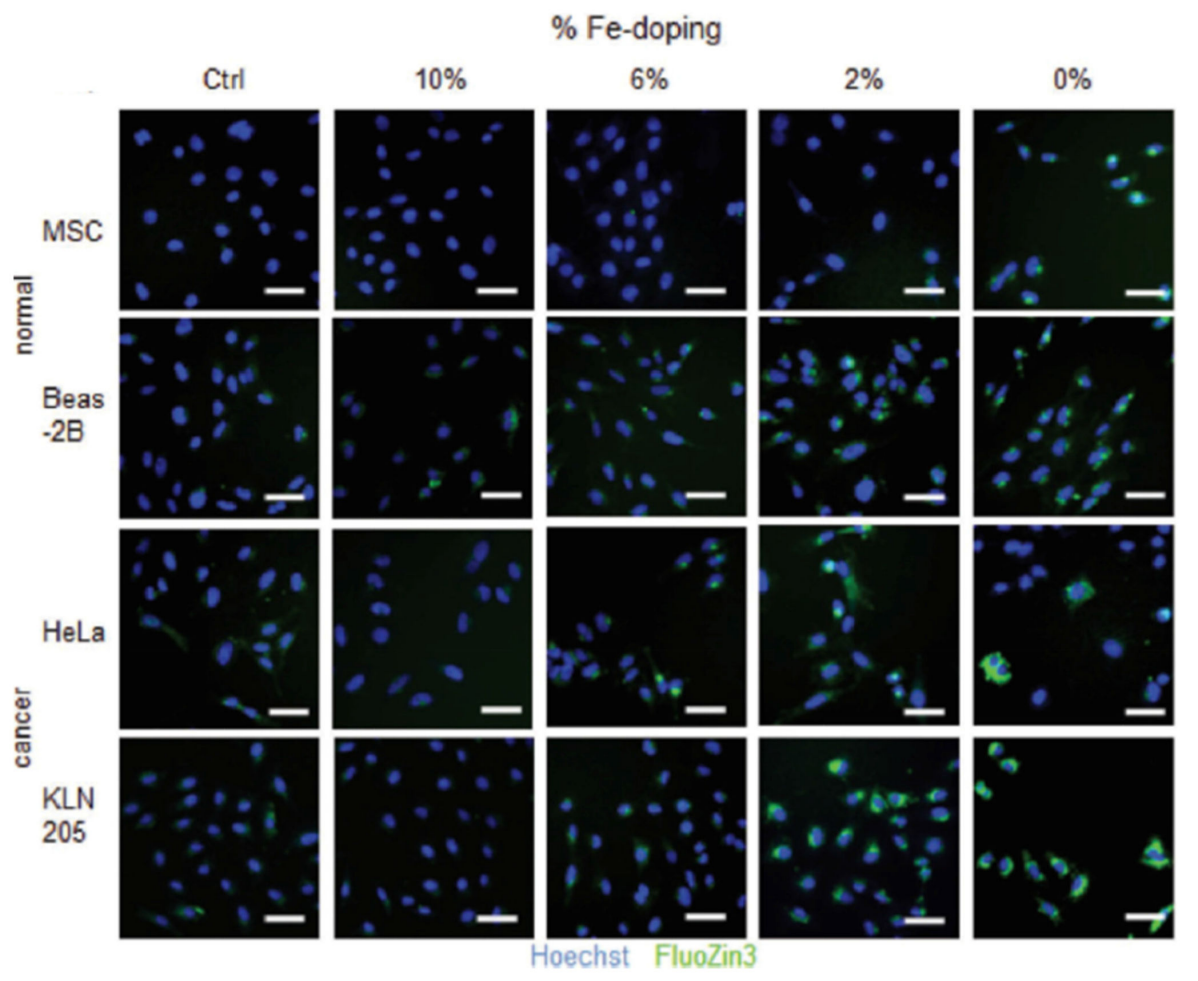
Fluorescence microscopy images of different cell lines exposed to differently Fe-doped ZnO nanoparticles (no nanoparticles (NPs) are present in the control samples). The green signal is related to Zn^2+^ free ions (FluoZin3-AM binds to ions and have a green fluorescent emission), the blue signal is related to the presence of cell nuclei. Less doped ZnO nanoparticles lead to a higher concentration of free zinc ions. Adapted from ref. [204].

In another work, Fe-doped ZnO nanoparticles, synthesized by flame spray pyrolysis, were tested in terms of cytotoxicity [204]. The authors analyzed different Fe doping levels to find the best one, -which allowed the selective killing of tumoral celis. The cytotoxicity assays were performed on normal murine mesenchymal stem cells (MSCs), human bronchial epithelial cells ŧBeas-2B), cancer murine lung squamous carcinoma cells (KLN-205) and human cervical cancer cells (HeLa. cells. The experimental results revealed that NPs with low doping levels were more efficient at damagtng tumoral cells rather than the healthy cells. Furthermore, the amount of Zn^2+^ released, measured by FluoZin-3 staining coupled with fluorescenee microscopy (Figure 8), was reduced by increasing the Fe doping.

In detail, 2 wf.% Fe doping was considered lhe best choice to achieve selectivity between tumoral and normal cells. This finding was further confirmed by co-culture cells experiments, finding an increased toxicity against cancer cells with respect to healthy ones. Moreover, undoped ZnO nanoparticles were tested as well, which are toxic for any cell line. On the other hand, 10 wt.% Fe-doped nanoparticles were biocompatible for both the cell typologies.

Fe-doped ZnO nanoparticles obtained with a similar synthesis method were tested in another work in vivo [205]. Hatching zebrafish embryos were taken as an indication of the toxicity of Fe-doped and undoped ZnO nanoparticles. Zebrafish were bred so as to achieve fertilization and the embryos were collected. Then, the embryos were treated with ZnO nanoparticles doped with different Fe levels, and the hatching rate was evaluated. The results showed that there were significant improvements with increased doping levels. Again, this was related to a decreased release of zinc cations. In the same work, it was also shown that iron doping can reduce the pulmonary inflammation and oxidative stress in rats and mice.

Despite the previous work being focused on the ability of a single dopant (iron) to stabilize the ZnO NPs stability, very promising results are found in terms of an increased cytocompatibility by using Fe-doped ZnO nanoparticles. This is corroborated by in vitro and in vivo tests, attributing the increased biocompatibility to a decreased level of zinc cations being released.

### 4.2 Antimicrobial Agents

**Figure 9:**
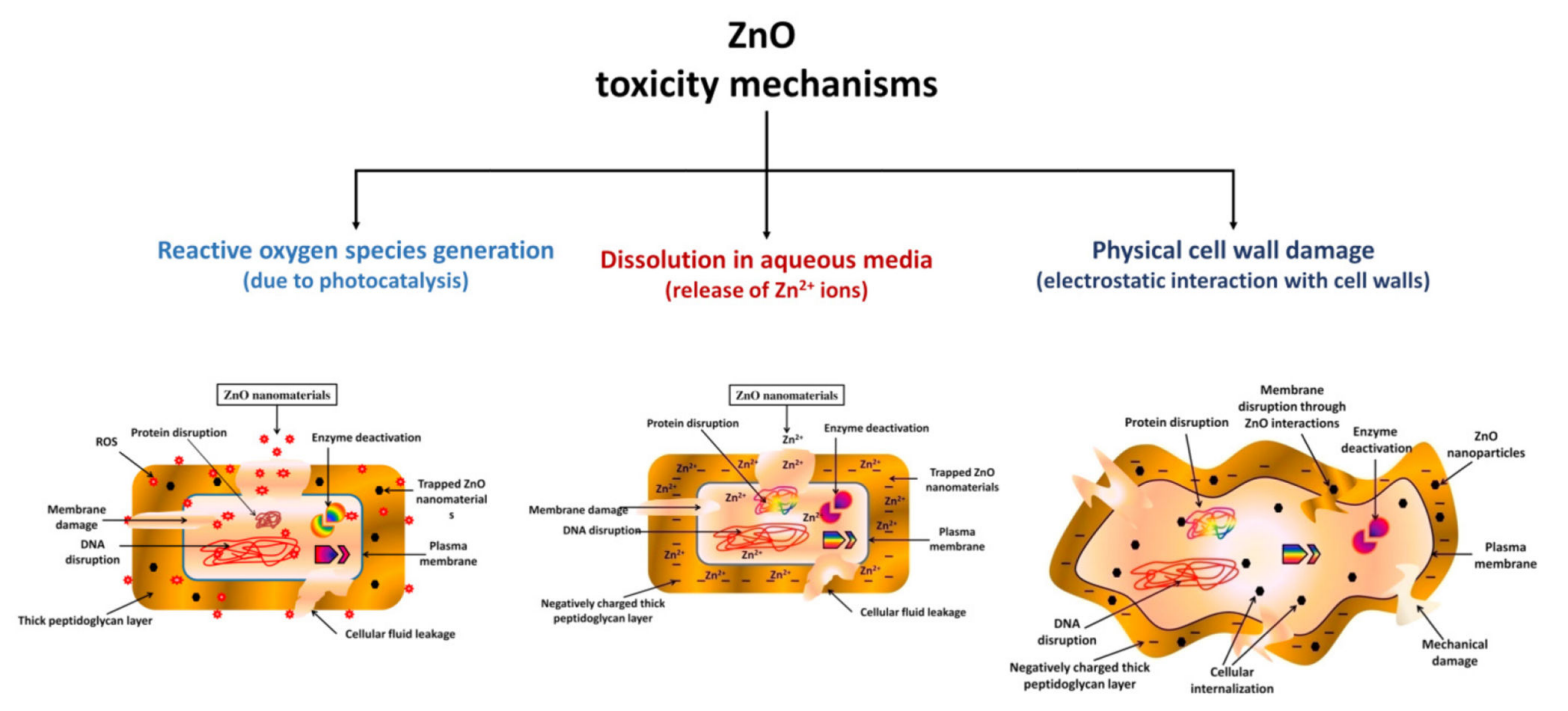
Schematic reporting the main ZnO toxicity mechanisms that make this material an effective antimicrobial agent Adapted from ref. [232].

Pure ZnO nanoparticles have been extensively used as antibacterial agents due to their photo-oxidizing and photocatalytic properties [216]. Its antimicrobial properties can be tailored in several ways: parameters like morphology, size, concentration, surface defects and functionalization all contribute to change the performances of ZnO nanoparticles in this field [216]. Despite the mechanisms through which ZnO induces bacterial and fungi death being still under debate, there are three main hypotheses, summarized in Figure 9.

The first one deals with ROS generation. As a matter of fact, ZnO is able to generate reactive oxygen species as a consequence of its photoexcitation from UV light in aqueous media [220]. The idea is that the electromagnetic radiation gives electrons lying in the valence band (VB) sufficient energy to jump to the conduction band (CB). The hole left in the VB can react with water, generating a hydroxylradical (^•^OH) and H^+^, while the CB electron can react with oxygen to generate a peroxide radical [231]. At this point, the following reactions can occur:

(4) $${}^{•}{\text{O}}_{2}+{\text{H}}^{+}\to {\text{HO}}_{2}^{•},$$

(5) $${\text{HO}}_{2}^{•}+{\text{H}}^{+}+{\text{e}}^{-}\to {\text{H}}_{2}{\text{O}}_{2},$$

This leads to the formation of hydrogen peroxide, which can d estroy bacteria by penetrating their membrane. If the generated ROS exceeds the capability of the brcterium to produce reducing agents able to contrast this phenomenon, oxidative stress phenomena are induced [232].

The second mechanism is based on the release of harmful zinc cations. Indeed, ZnO nanoparticles can disnolve releasing Zn^2+^ ions into the environment, affecting; the metabolism of bacteria and lea ding to dysfunctions of their enzymatic systems [216]. A work from Li et al. reported evidences of these effects [219]. ZnO nanoparticles, bulk ZnO and a solution of Zn^2+^ ions were analyzed against *E*. *coli*, finding similar toxicity curves and confirming tire negative influence that zinc cations may have on bacterial viability. However, this work further confirmed that ZnO toxicity is subjected to several intrinsic conditions like the size, porosity, and concentration of the particCes, coupled with external parameters like pH and the composition of the environment [216].

Finally, another possible mechanism that explains the antibacterial action of ZnO is the absorption of the nanoparticles into the bacteria membrane, leading to a physical disruption of the cell wall and ultimately to the microorganism’s death [233]. This is possible because ZnO nanoparticles typically pretent a positive Z-potential, i.e., a positively charged surface, while the bacteria surlace is characterized by a negative charge. Therefore, ZnO NPs are aitracted toward the bacterium, damaging its outer membrane. Moreover, if the particles are smaller than 10 nm, NPs’ internalization into the bacterium can also occur, damaging the internal components of the cell f232].

Additionally, it is worth mentioning that a different behavior can be found for different: kinds of microorganisms. Actually/, among bacteria there exist two main typologies, i.e., Gram-positive and Gram-negative ones, which show a different external cell membrane structure [216]. Therefore, it is clear that tests aimed at proving the antimicrobial mechanism of ZnO nano structures should be performed on both these biological systems: Gram-negative bacteria such as *Escherichia coli* (*E*. *coli*), and Gram-negative ones such as *Staphylococcus aureus* (*S*. *aureus*) [234,235].

Doping has been demonstrated as a useful tool to tailor the antimicrobial properties of ZnO nanoparticles. Indeed, the generation of antibacterial reactive oxygen species from the customization of the optical properties due to ZnO doping can be an advantage.

Some improvements in the photocatalytic properties and photoexcitation efficiency, which are the main mechanism through which antimicrobial ROS are generated in ZnO-based systems, were found in several works by using different doping agents [138,143,185,209].

For example, silver is a possible choice in terms of dopants which are useful for increasing the antibacterial activity of ZnO. The reason lays in the intrinsic bactericidal activity of silver ions against a wide spectrum of bacteria typologies [186]. As shown in the work of Demirel et al., ZnO structures with large surface area morphologies and doped with different Ag amounts were tested against different microorganisms (bacteria, yeasts and fungi) [233]. Disk diffusion tests highlighted that hexagonal ZnO particles were effective in contrasting microorganism growth when pure ZnO was used. However, Ag doping was found to be efficient in boosting the antibacterial properties (with an optimal doping value of 0.05 mol.%) against most of the tested biological species, resulting in a broad spectra antimicrobial device.

Another work reported the comparison between pure, gold- and silver-doped ZnO nanoparticles synthesized by a combustion method [134]. The photocatalytic efficiency resulting from the degradation of methylene blue under UV exposure was proven to be higher for the doped particles. In particular, Ag-doped ZnO NPs reached a 45% methylene blue degradation efficiency after 160 min against a 25% efficiency for Au-doped ZnO and 8% for undoped ZnO. This result was attributed to the noble metal clusters formed on the surface of ZnO nanoparticles during synthesis, which inhibited the recombination of the photogenerated electron/hole pairs. However, the antibacterial activity against *E*. *coli* and *S*. *aures* was lower for doped ZnO with respect to the pure counterpart. On the contrary, Ag doping was effective against *E*. *ashbyii*, even if a direct comparison with other doped ZnO systems was difficult because of the presence of dopant clusters rather than a correct inclusion of dopant atoms in the ZnO crystal lattice. In the work by Sharma et al. [165], ZnO particles were successfully doped, including Ag ions into the crystal lattice and a reduction of the minimum inhibitory concentration (MIC) was found for *S*. *aures* with respect to pure ZnO, in contrast to what was found in the previous work.

Silver doping was also compared to copper doping in terms of antimicrobial activity. Hydrothermally synthesized undoped, Cu- and Ag-doped ZnO nanoplates were tested against *E*. *coli*, *S*. *aureus* and *Salmonella typhi* by a well diffusion method in order to determine the MIC [152]. The results reported an increased bactericidal activity of both doped ZnOs, the result of a decreased MIC. Moreover, Ag-doped nanoplates were more efficient than Cu-doped ones. In this work, the authors also highlighted the role of light. The antimicrobial properties of the doped ZnO nanostructures were limited when the bacterial seeding and treatment processes were performed in dark conditions, suggesting that the photoexcitation of the semiconductor plays an important role in determining the antimicrobial activity of ZnO.

Cu-doped ZnO nanoparticles were analyzed in terms of antibacterial activity in another study. A green combustion method was followed to synthesize the nanoparticles, which were tested against *E*. *coli*, *S*. *aureus*, *Bacillus subtilis* and *Klebsiella* through the agar diffusion method [236]. Their photocatalytic activity was also evaluated by measuring the degradation of the Acid Black 234 (AB) organic dye under sunlight irradiation. Using the Cu-doped ZnO nanoparticles resulted in a more efficient dye degradation with respect to the pure counterpart as well as an inhibition of the bacterial growth for all the examined biological species. In particular, ZnO nanoparticles with higher levels of Cu doping showed enlarged inhibition zones with respect to a standard drug (cephradine). The reason behind the antibacterial activity of these nanoparticles was attributed to the permeation of Cu ions through the negatively charged bacterium cell membrane because of its electrostatic attraction.

Al-doped ZnO (AZO) nanorods were also proven to have superior antimicrobial properties with respect to pure ZnO [187]. The AZO nanorods showed a larger inhibition zone against *E*. *coli* and *E*. *hirae* (10.19 ± 0.04 mm and 10.20 ± 0.2 mm, respectively) with respect to pure ZnO (9.54 ± 0.08 mm and 9.62 ± 0.08 mm), and similar results with respect to the kanamycin drug (10.16 ± 0.07 mm and 10.17 ± 0.08 mm). This behavior was again attributed to the electrostatic interaction between the particles and the bacterial cell walls.

Iron doping was studied to improve the antibacterial ability of ZnO as well. In the work of Li et al. [122], Fe-doped ZnO nanoparticles synthesized by flame spray pyrolysis were tested against *B*. *subtilis*, *E*. *coli* and *Pseudomonas putida* through a high throughput bacterial viability assay able to evaluate the viability of bacteria through fluorescence signals. Interestingly, Fe doping did not impact in a major way on the antibacterial activity of ZnO, finding comparable results with respect to its pure counterpart. In another work, Fe- and Mn-doped ZnO nanoparticles were synthesized by a wet chemical method and analyzed in terms of photocatalytic activity before the antimicrobial tests [237]. The degradation of methylene blue under UV light was taken to identify the system with the best photocatalytic activity, which was 10% Fe-doped ZnO NPs. Disc diffusion assays reported that the 10%-doped system (both Mn- and Fe-doped) exhibited the highest antimicrobial activity against several pathogenic entities compared to lower doped (1%) systems and pure ZnO NPs. The reason for the improvement of both photocatalytic and antibacterial properties was related to the increased generation of ROS, and that Mn and Fe ions may lead due to the formation of additional states in the band-gap structure of ZnO.

Additionally, magnesium was proven to increase the antimicrobial performances of ZnO. It was found that different Mg doping levels change the photocatalytic activity and rhodamine B degradation of the doped ZnO nanoparticles, finally influencing their antibacterial activity against both Gram-positive and Gram-negative bacteria [138]. The photocatalytic activity was found to increase with the doping level, reaching a maximum in the case of 7.5% Mg doping, and then decreasing for a further increase of the doping amount. The enhancement was attributed to the enlarged surface area of the ZnO NPs and to the oxygen vacancies induced by doping. For the antibacterial activity, disc diffusion analyses gave, as a result, an increment of the inhibition zone with the increase of the doping level. Another work reports the use of Mg-doped ZnO NPs loaded with an antibiotic drug [166]. An enhanced antimicrobial activity was found, and this behavior was attributed to the destabilization of the cell wall induced by the nanoparticles and their consequent increased permeability, which allowed the antibiotics to penetrate the bacterium membrane more efficiently.

Rare earth elements were also proposed as dopants to enhance the antibacterial properties of ZnO. In the work of Karunakaran et al., Ce-doped ZnO nanoparticles were tested first as a photocatalyst for cyanide photooxidation and then as a antimicrobial agent against *E*. *coli* [209]. The authors reported an increased bactericidal activity in dark conditions for the doped ZnO NPs which was related to the physical attachment of the particles onto the bacteria membranes. Other examples are terbium and gadolinium [238]. Both were tested in the work of Daksh et al., in terms of photocatalytic activity, evaluating the efficiency in victoria blue degradation and in terms of the antibacterial activity against *S*. *Aureus* [238]. In both cases, increased performances were found with respect to pure ZnO.

The studies mentioned before highlight that doping is a valuable tool to enhance the antibacterial activity of ZnO nanoparticles. Both Gram-positive and Gram-negative bacteria appear to suffer the presence of doped nanoparticles since no particular differences are generally found in the two bacteria typologies. Moreover, the mechanism through which the doping can increase the toxicity against bacteria appears slightly different among the various dopants. Many authors reported an increased photocatalytic activity which led to an increased ROS generation. In other cases, the increased bactericidal action was ascribed to a more efficient attachment of the nanoparticles to the cell membrane. The exact reason behind the improved antibacterial toxicity of doped ZnO nanoparticles is still unclear and further studies are required for each of the proposed dopants to assess the role of charge and the structural and electronic properties of doped ZnO on the antibacterial activity.

### 4.3 Nanotools for Photobiomaging

#### 4.3.1 Photoluminescence Properties

Pure ZnO has a wide band-gap of about 3.3 eV at room temperature and thus can only absorb UV light which is just 5% of the solar spectrum [239]. To improve the light absorption efficiency toward a more biologically-relevant visible-near infrared (vis-NIR) region, the modification of ZnO nanoparticles with impurities incorporated in the host lattice has been introduced. Actually, the stimulation of the photoluminescent response of doped ZnO NPs by using vis-NIR light, which does not damage tissues and organs, is expected to be particularly useful in bioimaging applications. Herein, to extend the emission range from the intrinsic band-gap of ZnO to the infrared region, doping with optically-active impurities like transition metals (TM) or rare earth (RE) elements has been reported even though TM and RE elements reveal different spectral properties [239]. For TMs, as the influence of the surroundings is stronger due to the weaker shielding of the 3d-shell by the 3s orbit, the spin-orbit coupling is weaker and the 3d-levels are broad. As a result, the energetic spacing varies with the host material and the electron-phonon interaction appears stronger than RE elements [240]. For RE elements, because of the strong shielding of the 4f-shell by the 5s2 and 5p6 orbits, the interaction with the surroundings is weak and almost all RE elements show intra-shell luminescence, i.e., the one least occupied, and unoccupied intra-shell levels are located in the band-gap of ZnO [240,241]. Thus, a very similar chemical behavior for all lanthanides in ZnO has been identified, whereas filling the d-shell in transition metals has a stronger influence on the outer shells and its bonding behavior in the ZnO lattice. To this end, there are various reports incorporating optically-active dopants in the ZnO lattice to change the band-gap value and increase the UV luminescence properties of pure ZnO [242–244]. Additionally, the optical parameters (band-gap) of the doped ZnO nanoparticles are summarized in Table 3. The band-gap values of the doped ZnO depend on several factors such as grain size, carrier concentration, lattice strain, size effect, oxygen vacancy and its related disorder-created localized defect states within the band-gap of ZnO, orbital hybridization between the dopant and the host band, etc. [245,246]. Depending upon these factors, red shift behavior (or a decrease in the band-gap) and blue shift behavior (or an increase in the band-gap) have been reported by several research groups (Table 3).

#### 4.3.2 Exploiting Room Temperature Ferromagnetism for Imaging

In recent years, diluted magnetic semiconductors (DMS) materials have attracted great research interest due to their potential combination of both semiconductive and magnetic properties. The application of ZnO-DMS, exploiting the electron charge associated with the intrinsic spin of the electron, has been noticed in spintronic devices [107,247]. It has been predicted in theory as well as proven in practice that ZnO nanoparticles doped with transition metals (TMs) or rare earth (RE) elements can result in a DMS material with a high Curie temperature, comprising ferromagnetic properties at room temperature [248–250]. The magnetic properties of TM or RE elements are implemented by the magnetic moments generated due to the presence of unpaired electrons in the outermost 3d or 4f orbital, respectively, although the nature of the magnetic moments of TM and RE elements are quite different. For instance, TMs have a small total magnetic moment per atom, as the outermost 3d electrons in TMs are exterior and delocalized, and their orbital momentum is frequently zero [248]. On the other hand, in RE elements, the peripheral 4f electrons are localized, having indirect exchange interactions among 5d or 6s conduction electrons. Therefore, RE elements can be expected to have a high total magnetic moments per atom owing to its high orbital momentum than that of TMs [113,251]. Various TMs such as Fe, Co, Cr, Mn, and Ni, and RE elements such as Eu, Tb, La and Gd have been exploited as dopants for creating doped ZnO NPs DMS. The magnetic parameters of the doped ZnO NPs are summarized in Table 3. Besides, there are few reports showing an enhanced ferromagnetism by exploiting Co-doped ZnO NPs [252–254]. The doped ZnO NPs can also be exploited as magnetic resonance imaging (MRI) contrast agents either to increase the signal intensity on T1-weighted images or to reduce the signal intensity on T2-weighted images [255–257]. Moreover, some research groups have reported Gd-doped ZnO quantum dots (QDs) as efficient dual modal fluorescence and MRI nanoprobes [163,258].

### 4.4 Doped ZnO as Therapeutics against Cancer

ZnO nanoparticles present interesting properties useful for effectively fighting cancer as well. ROS generation and the ZnO particles dissolution are the main mechanisms responsible for cell and bacterial death. Therefore, it can be potentially toxic for tumoral cells.

**Figure 10:**
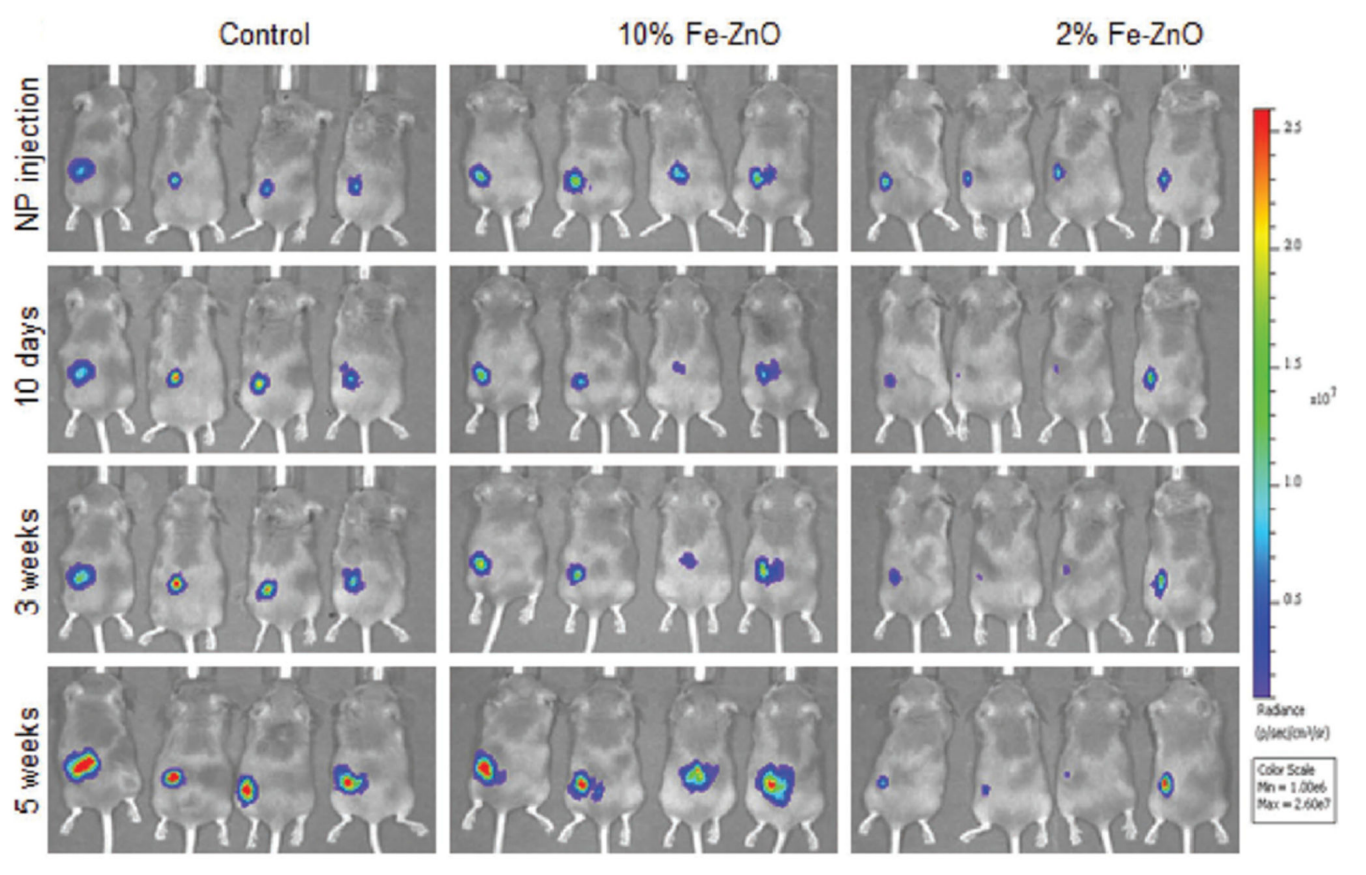
Tumor growth trend in mice at different times and differently doped ZnO nanoparticles.Tumor growth is reduced with 2% Fe-doped ZnO nanoparticfes. Adapted from ref. [204].

For ZnO dissolution, the role of Fe doping as a stabilizer for the ZnO crystal lattice has been highlighted in Section 4.1, leading to a decreased dissolution rate of the nanoparticle. This has been proven to also inhibit tumoral cell growth. As a matter of fact, tumoral cells appeared to be more sensitive to the presence of ZnO than healthy ones [218,283]. By finely tuning the dissolution rate of ZnO nanoparticles with Fe doping, it was possible to reduce the toxicity to an optimal value so as to be harmful for cancer cells without damaging healthy cells [204]. The promising results obtained in in vitro tests gave the basis to perform a preclinical study on a rodent model for evaluating the antitumor response: pure 2 wt.% and 10 wt.% Fe-doped ZnO nanoparticles were tested in mice, finding again a reduced amount of free zinc ions being released, and a decreased tumor growth in the presence of the Fe-doped ZnO NPs. In particular, the tumor (KLN-205) was inoculated subcutaneously in mice and, after 10 days of treatment with pure ZnO NPs, mice displayed signs of toxicity to force the authors to stop the experiment with pure ZnO for ethical reasons. Figure 10 shows the growth of the tumor for the 2 wt.% and 10 wt.% Fe-doped ZnO NPs and the control (saline solution) treatments. As it can be seen in Figure 10, the 2 wt.% doped ZnO nanoparticles effectively inhibited the tumor growth, different to the control and the 10 wt.% doped ZnO NPs treatments.

**Figure 11:**
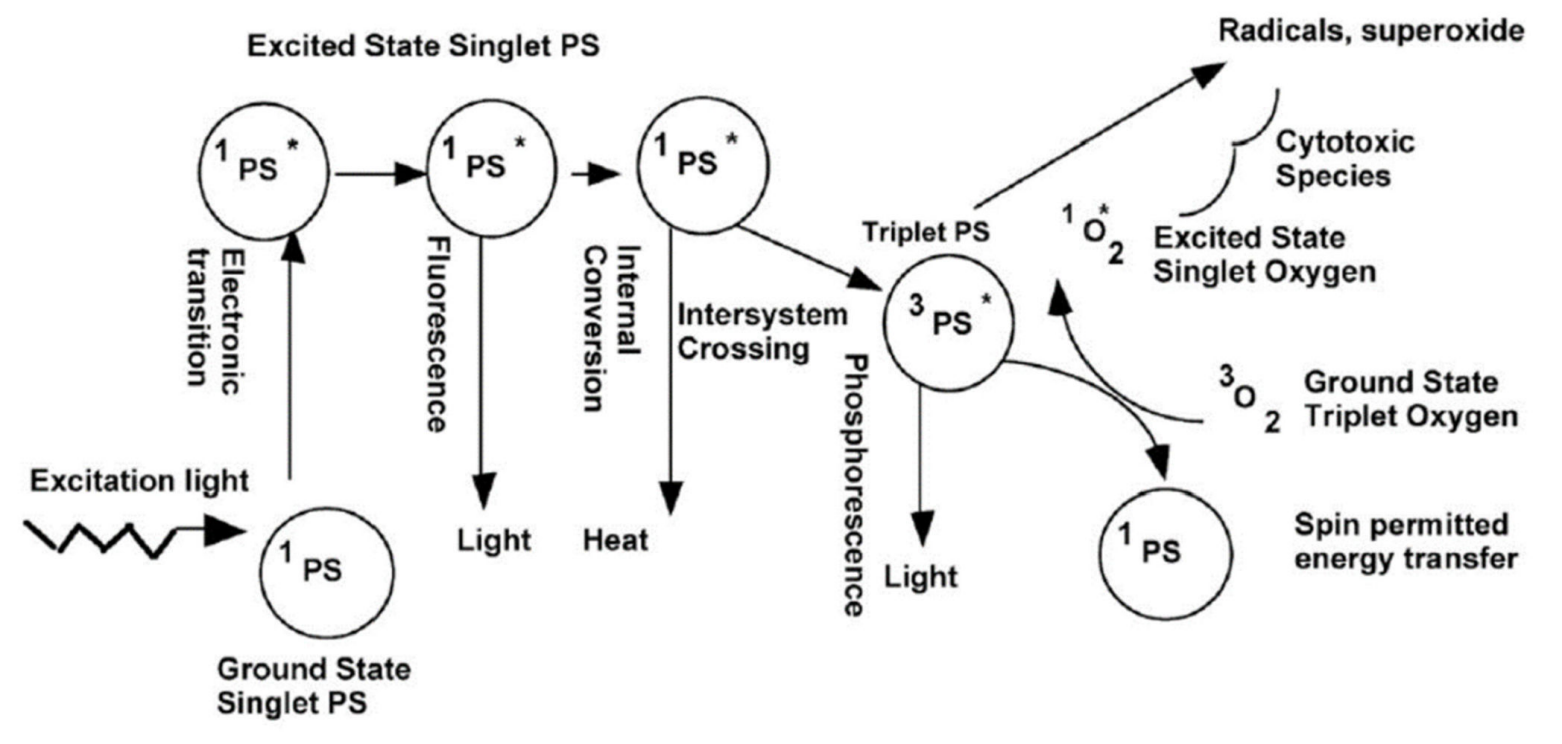
Photodynamic therapy mechanism. The photosensitizer (PS) is excited by external radiation, the excited electron may decay through different phenomena which may end in the generation of cytotoxic species, like ROS. Adapted from ref. [284].

Concerning ROS generation, ZnO NPs was successfully applied in photodynamic therapy. The idea behind this technique is to exploit the interantion between a photosensitizer (the semiconducting ZnO NP) and electromagnetsc radiation in order to generatn cytotoxic species (like ROS) that kilS cells and destroy tissues [284], with the mechanisms eepsnted schematically in Figure 11. The photosensitizee should be internalized inside the cell and then exposed to light. By photoexcitation, it is possible to induce the electronic transition from the valence to the conduction bend, which allows the formation oh ROS, as already described in Section 4.2.

In the work of Hackenberg et al., the role of ZnO nanoparticles on the viability of tumoral and healthy cells was investigated [285]. Pure ZnO nanoparticles were tested on a human head and neck squamous cell carcinoma (HNSCC) cell line and on oral mucosa cells (pOMC). Different concentrations of ZnO NPs and the exposition time to UV light were explored on cell cultures internalizing the particles, and cell viability was evaluated through an MTT assay. The results showed that a high concentration (20 μg/mL) of nanoparticles reduced the viability for all the cells (~70% of the survival percentage without UV exposure), while lower concentrations (up to 2 μg/mL) resulted in more toxicity toward cancerous cells when coupled with longer times of UV exposure (at a 2 μg/mL concentration of NPs and 15 min of UV exposure, ~40% of the survival percentage for cancerous cells vs. 90% for normal cells). Even if the exposure to UV light highlighted the important role played by the photocatalytic activity of ZnO NPs against cancer cells, the cytotoxicity observed at high concentrations in the absence of UV exposure also suggested the presence of a secondary mechanism.

ZnO nanoparticles for photodynamic therapy were investigated also by Ancona et al. [92]. In this case, ZnO particles were coated by a lipid layer in order to increase the dispersion of the nanoparticles in biological media and to improve cellular uptake, and were then tested as photosensitizers against human epithelial carcinoma cells (HeLa). The lipid-coated nanoparticles were successfully internalized in the cells and an increased cytotoxicity was found with an increase of the concentration of NPs coupled with UV irradiation, confirming the interesting antitumoral properties of bare ZnO.

In the previous sections of this review, several works reporting an increased photocatalytic activity of doped ZnO nanoparticles were described. This aspect can be extremely useful in enhancing NPs cytotoxicity against tumoral cells, in a similar manner to what has been demonstrated for antimicrobial applications.

Lithium-doped ZnO nanoparticles were proposed as generators of singlet oxygen for this purpose [144]. A physical characterization of these nanoparticles was provided, reporting an increase of the band-gap energy and a slight blue shift of the exciton energy. Moreover, an increased generation of singlet oxygen was clearly observed for the increasing doping levels, suggesting its use in photodynamic therapy.

**Figure 12:**
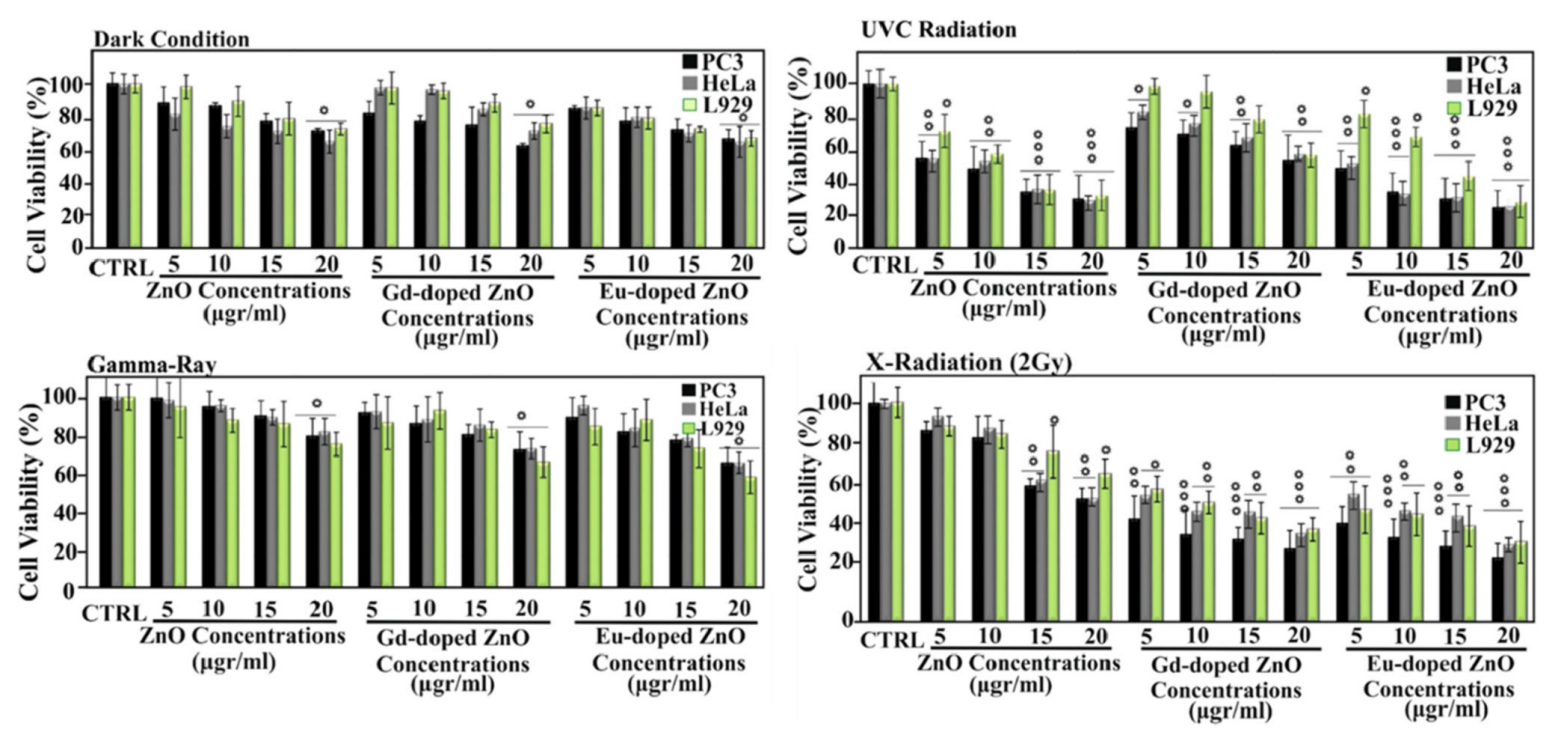
Cell viability of different cell lines exposed to different illumination sources and different concentrations, incubated with pure, Gd-doped and Eu-doped ZnO nanoparticles. Adapted from ref. [164].

In this sense, gadolinium and europium were also considered. In the work by Ghaemi et al., these two chemical species were proposed as dopant ions in ZnO nanoparticles and their influence was analyzed in terms of their cytotoxic behavior against normal (L929 fibroblast cells) and tumoral (Hela cervical cancer cells and PC3 prostate cancer cells) cells [164]. In particular, Gd- and Eu-doped ZnO NPs were internalized into cells and the cell cultures were exposed to UV, X-ray and γ radiations. Then, the cells viability was evaluated by an MTT assay, giving the results reported in Figure 12. In dark conditions, the NPs only slightly decreased the cells’ viability with the increase of the concentration, demonstrating that Gd- and Eu-doped ZnO nanoparticles did not induce considerable cell death. However, when UV exposure was considered, the cells viabilities heavily reduced for all the doped and undoped-treated cell cultures, suggesting the importance of the photoexcitation of the ROS production. Another important aspect was a remarkable selectivity in killing only cancerous cells if intermediate concentrations of doped ZnO particles (10 μg/mL) were used. Cell lines incubated with ZnO NPs were also exposed to X-rays and γ-rays. While γ-rays did not give important changes in the viability of cells with respect to dark conditions, X-rays at 2 Gy were extremely effective at killing cells, with doped ZnO NPs in particular, perhaps because of a higher ROS generation.

**Figure 13:**
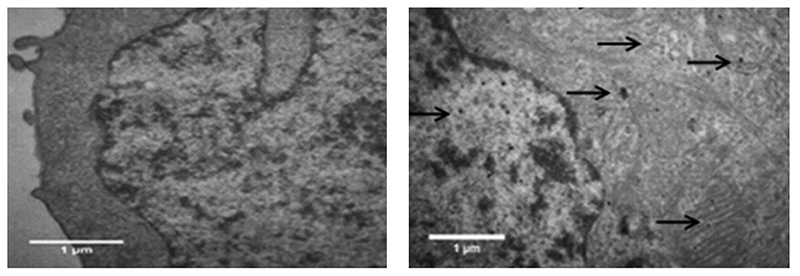
TEM images showing the internalization of pure (**a**) and Gd-doped (**b**) ZnO nanoparticles in lung carcinoma (SKLC-6) cells. Adaptedfrom ref. [286].

Gadolinium doping coupled with X-ray r adiation was tested also in an other study, where Gd-doped ZnO nanoparticles were administered to lung carcinoma cells (SKLC-6) and irradiated with megavoltage Xfrays [186]. Nanoparticles with a dimension of 9 nm wese successfufly internalized in the cell bodies as shown in Figure 13. A dose-dependenf effect under X-rays irradiation was observed also in this case; tha cells viahility decreased when the NPs concentration was increased, reaching ~20% of cell viability with respect to the control at 100 μg/mL of the NPs concentration, as well as a reduction in the DNA repair effic iency.

Additionally, Mg-doped ZnO nanoparticles were proven to induce cell death in human breast cancer cells (MCF-7), and AO & EB staining couplad with fluorescence microscopy showed that the main way through which cell deed was apoptosis [166].

Cu-doped ZnO nanoparticles synthesized by a green methad demonstrated simtlar anticancer properties [236]. The aomparis on on the cell viability between pure ZnO NPs, Cu-dopod ZnO NPs and doxoruticin showedsuperior cytotoxic effects pf the doped NPs against breast cancer cells. The proposed mechanism for cell dealh was the penetration of the small particles thro ugh the cell barrier.

Despite ZnO being already proposed as a therapeutic agent against cancer, doped ZnO nanoparticles still do not have a large literature pool through which the mechanism of toxicity can be stated univocally. Many studies have reported an increased cytotoxicity in concomitance with increased photocatalytic and antibacterial activities. Other works focused on the stability of the ZnO particle to tune specifically the antitumoral effect. Even if further studies are required, the results herein described clearly show that doped ZnO can be a powerful weapon to fight cancer.

## 5 Conclusions

In summary, doping represents a valuable tool to extend the use of ZnO NPs in nanomedicine. Wet chemistry approaches based on sol-gel, hydrothermal routes and combustion methods have been largely used to dope ZnO NPs with various elements including rare earth (REs) elements and transition metals (TMs). It is found that RE elements have been more extensively considered to improve the photocatalytic and optical properties, but also to confer on ZnO NPs magnetic and improved electromechanical behaviors. Overall, doping ZnO NPs with TMs was mainly considered to give ZnO unprecedented ferromagnetic properties.

Doped ZnO NPs have been successfully proposed as therapeutic agents against cancer and showed enhanced antibacterial properties against both Gram-positive and Gram-negative bacteria. The mechanism through which doping can increase ZnO NPs toxicity against cancer cells and bacteria is still controversial and not univocally stated. In some cases, it was ascribed to the improved photocatalytic activity and increased generation of harmful ROS. In other cases, the increased bactericidal action was due to a more efficient attachment of the nanoparticles to the cell membrane. Even if further studies are required, the results described herein clearly highlight that doped ZnO NPs can be a powerful weapon to fight bacterial infections and treat cancer. Doping ZnO NPs with TMs also represents a powerful approach to acquire new bioimaging properties which have been successfully applied to magnetic resonance imaging applications. Promising results were also found in terms of an increased cytocompatibility by using Fe-doped ZnO nanoparticles due to the reduced and even more controllable release of cytotoxic zinc cations.

## Figures and Tables

**Table 1:** Doped ZnO structural properties.

Ref.	Dopant Element	Doping Level	(Ox. State) Ionic Radius [pm]	*a*	*a_D_*-*a_0_* ^1^	*c*	*c_D_*-*c_0_* ^2^	Unit Cell Volume
[170]	Bulk ZnO	-	(+2)74	3.2500 Å		5.2047 Å	-	47.609 Å^3^
[116]	La	5% mol	(+3)103	3.2497 Å	−1.3 × 10^−3^ Å	5.2058 Å	−3.0 × 10^−3^ Å	47.610 Å^3^
[171]	Ce	1% mol	(+3) 101	3.2503 Å	−1.6 × 10^−3^Å	5.2058 Å	−6.2 × 10^−3^ Å	47.629 Å^3^
[172]	Nd	5% mol	(+2) 129, (+3) 98	3.2495 Å	12.9 × 10^−3^ Å	5.2058 Å	20.7 × 10^−3^ Å	47.605 Å^3^
[150]	Eu	5% mol	(+2) 117, (+3) 95	3.251 Å	2.0 × 10^−3^ Å	5.209 Å	4.0 × 10^−3^ Å	47.693 Å^3^
[157]	Gd	5% mol	(+3) 93 (+2)79,(+3)	3.2735 Å	20.6 × 10^−3^ Å	5.2128 Å	2.6 × 10^−3^ Å	48.375 Å^3^
[173]	V	5% mol	64, (+4)58,(+5)54	3.2522 Å	0.5 × 10^−3^ Å	5.2075 Å	−1.0 × 10^−3^ Å	47.699 Å^3^
[126]	Mn	5% at.	(+2)81,(+3)72 (+4)67,(+7)60	3.2520 Å	2.2 × 10^−3^ Å	5.2093 Å	3.0 × 10^−3^ Å	47.710 Å^3^
[174]	Fe	5.09% mol	(+2)75,(+3)69	3.2536 Å	2.3 × 10^−3^ Å	5.2093 Å	11.1 × 10^−3^ Å	47.757 Å^3^
[175]	Co	5% mol	(+2)79,(+3)68	3.2503 Å	−2.0 × 10^−3^ Å	5.2059 Å	−0.8 × 10^−3^ Å	47.629 Å^3^
[176]	Cu	5% at	(+1)91,(+2)87	3.2494 Å	−0.2 × 10^−3^ Å	5.2054 Å	−0.4 × 10^−3^ Å	47.598 Å^3^
[152]	Ag	5% mol	(+1)129,(+2) 108	3.2579 Å	3.6 × 10^−3^ Å	5.2220 Å	3.7 × 10^−3^ Å	48.000 Å^3^
[144]	Li	5% at	(+1)90	3.225 Å	−30 × 10^−3^ Å	5.162Å	−50 × 10^−3^ Å	46.495 Å^3^
[138]	Mg	5% mol	(+2)86	3.2585 Å	−3.6 × 10^−3^ Å	5.2181 Å	−7.5 × 10^−3^ Å	47.982 Å^3^

1 Relative variation of parameter a of doped ZnO (a_D_) with respect to the pure counterpart evaluated in the paper (a_0_).

2 Relative variation of parameters of doped ZnO (c_D_) with respect to the pure counterpart evaluated in the paper (c_0_).

**Table 2:** Dopant ions and their respective precursors for the synthesis of ZnO nanoparticles by wet chemical methods.

Dopant Element	Dopant Precursors	Doping Level	Solvent	Particles Dimensions	Ref.
La	LaCl_3_·7H_2_O	5% mol	H_2_O^a^	123 nm ^1^	[116]
Ce	CeCl_3_·7H_2_O	1% mol	H_2_O^a^	20–30 nm ^1^	[171]
	Ce(NO_3_)_2_·6H_2_O	0.1–5% mol	H_2_O^b^	70–85 nm ^1^	[112]
Nd	Nd(NO_3_)3·6H_2_O	5% mol	H_2_O^a^	101 nm ^1^	[172]
Sm	Sm(NO_3_)_3_ ·6H_2_O	1–4% mol	H_2_O^a^	35 nm ^1^	[193]
Eu	EuCl_3_·6H_2_O	5% mol	H_2_O ^a^	79 nm ^1^	[150]
	Eu(NO_3_)3·5H_2_O	5% mol	CH_3_OH ^a^	9 nm ^2^	[164]
Gd	Gd(NO_3_)3·6H_2_O	5% mol	CH_3_OH ^a^	9 nm ^2^	
	Gd(CH_3_CO_2_)_3_	2–30% mol	CH_3_CH_2_OH	4 nm^2,3^	[163]
V	NH_4_VO_3_	1% mol	H_2_O ^a^	47 nm ^2^	[194]
Mn	Mn(NO_3_)_2_	0.5–3% mol	CH_3_CH_2_OH	50–120 nm ^2^	[183]
	MnCl_2_ ·4H_2_O	1–5% mol	CH_3_OH	100 nm ^2^	[126]
Fe	Fe(SO_4_)·7H_2_O	3–7% mol	H_2_O^a^	15–35 nm ^2^	[195]
	FeCl_3_	1–10% mol	H_2_O^a^	9–15 nm ^2^	[162]
	Fe(NO_3_)_3_	2–6% mol	H_2_O^a^	~250 nm ^4^	[196]
Co	Fe(NO_3_)_2_ ·6H_2_O	1–10% mol	H_2_O	25–50 nm ^2^	[184]
	CoCl_2_	5–10% mol	H_2_O ^a^	Various morphologies	[175]
Ni	NiCl_2_·6H_2_O	3% mol	CH_3_CH_2_OH ^a^	25–40 nm ^1^	[159]
Cu	CuCl_2_·2H_2_O	0.5–30 at.%	H_2_O^b^	~250 nm ^4^	[176]
Ag	AgNO_3_	5% mol	H_2_O ^a^	80 nm × 350 nm ^5^	[152]
Li	Li(CH_3_CO_2_)_3_·2H_2_O	3-5 at.%	TREG (C_6_H_14_O_4_)	~250 nm ^4^	[144]
Mg	Mg(NO_3_)2·6H_2_O	5% mol	H_2_O ^a^	62 nm	[166]
Al	Al(NO_3_)3·9H_2_O	15% mol	H_2_O ^a^	~60 nm ^1^	[187]

a NaOH as base

b NH_4_OH as base

1 Rod’s mean diameter.

2 Spherical particles’ diameters.

3 TMAH (tetramethylammonium hydroxide) and oleic acid were also added in the synthesis procedure.

4 The dimensions have been estimated by electron microscopy images.

5 Nanoplates’ thicknesses and diameters.

**Table 3:** Doped ZnO: optical and magnetic properties.

Ref.	Dopant	Dopant Concentration	Bandgap (eV)	Dopant Concentration	Saturation Magnetization (emu/g)
[259–261]	Cu	0, 10%	3.35, 3.30, respectively	0.05–0.20 mol.%	0.011–0.063
[180,262]	Fe	Zn_1-x_Fe_x_O (x = 0, 0.01, 0.04, 0.05, 0.06)	3.243, 3.236, 3.216,3.197, 3.195, respectively	x = 0.20	1.74
[263]	Cr	0.00 to 4.63 at.%	from 3.26 to 3.15	2.49 at.%	4.86
[175]	Co	0, 5,10 at.%	3.10, 3.17, 3.24, respectively	5 at.%, 10 at.%	1.42,1.75
[264–267]	Mn	0, 3, 5,10,15 mol.%	3.31, 3.35, 3.38, 3.40, 3.42, respectively	3.3 mol.%, 4.2 mol.%	0.00123, 0.015
[268]	Ni	Zn_1-x_Ni_x_O (x = 0, 0.05)	3.28, 3.32, respectively	x = 0.05	2.9-2.8
[269,270]	Al	0, 2 at.%	3.07, 3.12, respectively	0.03 at.%	0.012
[138,271]	Mg	0, 2.5%, 5%, 7.5%	3.36, 3.27, 3.13, 3.04, respectively	3%	1.05 × 10^−3^
[272]	Nd	ZnO, Zn_0.97_Nd_0.03_O	3.34, 3.12, respectively	x = 0.03	0.67
[273,274]	Sm	0,1, 3, 5 mol.%	3.27, 3.25, 3.10, 3.05, respectively	0%–8%	0.45, 0.363, 1.694,3.613 and 2.197 emu/cm^3^
[275,276]	Eu	0,1, 3, 5 mol.%	3.18, 3.05, 3.00, 2.94, respectively	10%	0.040
[277]	Tb	Zn_1-x_TbxO (x = 0, 0.02, 0.05, 0.1)	3.35, 3.31, 3.30, 3.28, respectively	x = 0, 0.02, 0.05, 0.1	0.0042, 0.0276, 0.0359, 0.0519
[117,278]	Gd	0, 3, 6%	2.71, 2.74, 2.98, respectively	1.1%, 3.5%, and 5.1%	0.0001, 0.05, 0.0032
[279,280]	La	1, 5 wt.%	3.12, 3.18, respectively	0, 1 mol.%	0.102,0.232
[281,282]	Ce	0,1, 3 and 5 at.%	3.21, 3.10, 3.08, 2.96, respectively	0,0.96,1.96, 2.52 and 3.12 at.%	1.895 × 10^−3^, 31.612 × 10^−3^, 26.818 × 10^−3^, 26.136 × 10^−3^, 23.608 × 10^−3^
